# Measuring cue-elicited responding in the context of gaming and online shopping: Validity and reliability of a short Pavlovian-to-instrumental transfer paradigm

**DOI:** 10.1038/s41598-025-27859-0

**Published:** 2025-12-29

**Authors:** Anna M. Schmid, Patricia Schaar, Ferdinand Gut, Lilli Herbelßheimer, Niklas Meurer, Charlotte Arend, Damla Seck, Patrick Ort, Astrid Müller, Matthias Brand, Sabine Steins-Loeber

**Affiliations:** 1 Department of Clinical Psychology and Psychotherapy, University of Bamberg, Bamberg, Germany; 2 Department of Psychosomatic Medicine and Psychotherapy, Hannover Medical School, Hanover, Germany; 3 Department of General Psychology: Cognition, Faculty of Computer Science, University of Duisburg-Essen, Duisburg, Germany; 4 Center for Behavioral Addiction Research (CeBAR), Center for Translational Neuro- and Behavioral Sciences, University Hospital Essen, University of Duisburg-Essen, Essen, Germany; 5 Erwin L. Hahn Institute for Magnetic Resonance Imaging, Essen, Germany

**Keywords:** Pavlovian-to-Instrumental transfer, Retest reliability, Habits, Problematic gaming, Problematic online shopping, Human behaviour, Addiction

## Abstract

Cue-elicited habits are considered relevant in the development and maintenance of addictive behaviors. To investigate this mechanism in online shopping and gaming, this study developed and evaluated a short Pavlovian-to-instrumental transfer (PIT) paradigm with gaming- and shopping-related cues and a devaluation procedure. Additionally, the retest reliability of this short PIT paradigm was assessed. A sample with game use (*N* = 32) and a sample with use of shopping websites (*N* = 31) performed the PIT paradigm twice and an Approach-Avoidance Task in between. During both sessions of the PIT paradigm, either the gaming- or shopping-related reward (depending on the sample) was devalued by eliminating its availability. While gaming-related cues enhanced responding for the gaming-related reward (gaming PIT effect), shopping-related cues triggered responding for the shopping-related reward (shopping PIT effect). This cue-elicited responding was reduced, but not eliminated, after devaluation, which might indicate habit-like responding. Furthermore, subgroup analyses revealed similar responding towards cues displaying favorite and non-favorite games/shopping websites. The magnitudes of the PIT effects demonstrated moderate stability. Although replication in clinical samples and over longer time intervals is warranted, our PIT paradigm may enable a reliable and economic assessment of cue-elicited responding in the context of gaming and online shopping.

**Supplementary Information:**

The online version contains supplementary material available at 10.1038/s41598-025-27859-0.

## Introduction

Conditioned cues play a relevant role in guiding everyday behaviors but can also contribute to the development and maintenance of maladaptive behaviors like addictions^1,2^. In the course of addictions, reactions to drug-related stimuli may increasingly manifest themselves as habit-like and finally even compulsive behaviors, meaning that drug-seeking occurs even in the face of aversive consequences^1,2^. This mechanism is not only discussed for substance use disorders, but has also been proposed for behavioral addictions^3^. Problematic use patterns resembling addictive behaviors have been described, among others, with regard to gaming and (online) shopping^4^. While gaming disorder has been included as a disorder due to addictive behaviors in the ICD-11^5^, the classification of buying-shopping disorder as a potential behavioral addiction is still subject of debate^4^, although compulsive buying-shopping disorder is mentioned as an example for other specified impulse control disorders in the ICD-11^6^.

Cue-elicited responding can be studied with Pavlovian-to-instrumental transfer (PIT) paradigms^7,8^. Typically, a PIT paradigm consists of three phases: During the Pavlovian training, an association between initially neutral stimuli (S) and rewarding outcomes (O) is established^9^. In a separate instrumental training phase, which can be conducted before or after the Pavlovian training, individuals learn that different instrumental responses (R) are associated with different rewarding outcomes (O)^9^. In the transfer phase, these responses are then tested in the presence of the conditioned stimuli of the Pavlovian training^7,8^. If stimuli associated with a certain outcome increase responding for this outcome, this is called a specific PIT effect^7,8^.

Most of the research on PIT effects in addictions has focused on substance use disorders ^e.g.,10–13^, however, recently, the PIT paradigm has also been used to study behavioral addictions^14–18^. This research has provided evidence not only for shopping- and gaming-related specific PIT effects^14–17^, but also for an association between the magnitude of gaming-related PIT effects and risky gaming behavior^15^ or gaming disorder^14^.

While cue-elicited responding has been repeatedly observed in PIT paradigms, its nature, i.e., whether it is more in line with habitual or goal-directed behavior, is still a matter of debate. According to the habitual account, the PIT effect is the result of a S-O-R chain^9,19–22^, which builds upon S-O and bidirectional R-O/O-R associations formed during Pavlovian and instrumental training^9,19^. During the transfer phase, the conditioned cue is assumed to activate the representation of the associated outcome, which then triggers the instrumental response linked with this outcome^9,19,21^. Importantly, the conditioned cue is considered to elicit only the sensory properties of the associated outcome, albeit not its current motivational value^9,21^. The goal-directed account, in contrast, considers the PIT effect to present a controlled action, which reflects individuals’ propositional beliefs concerning the task contingencies^9,23–25^. In particular, individuals may believe that the Pavlovian cue signals which outcome is available and may hence expect the congruent response to be more successful^9,23–25^.

To differentiate between habitual and goal-directed behaviors, researchers usually work with devaluation procedures, in which the reward value of one of the outcomes is decreased, for example by taste aversion ^e.g.,26^ or consumption to satiety ^e.g.,22^. If participants continue responding for the devalued outcome, the behavior is considered to be habitual^27^. Up to date, the evidence regarding the nature of the PIT effect is mixed, with some studies demonstrating the PIT effect to be sensitive to devaluation and other studies reporting it to be unaffected by a change in outcome value^9^. In two recent studies, which applied a PIT paradigm with gaming- and shopping-related cues and rewards, devaluation decreased but did not eliminate the PIT effect^15,16^, implying at least partly habit-like responding.

Hence, combining PIT paradigms with a devaluation procedure allows to measure not only cue-elicited responding in general but specifically habit-like responding in the presence of reward-related cues. In the following, we use the term *cue-elicited responding* when referring to the behavior measured with the PIT paradigm in general, regardless of its underlying mechanism, i.e., whether it is under habitual or goal-directed control. Regarding the PIT paradigm used in our study, the term is applied to describe behavior in both the part before and after devaluation. In contrast, we use the term *habit-like responding* when referring to continued responding for the devalued outcome after devaluation.

A common obstacle in studies using the PIT paradigm is that some individuals fail to acquire awareness of the stimulus-outcome associations during the Pavlovian training phase ^e.g.,10,14,17,28^. While research on individual characteristics affecting the acquisition of awareness has provided interesting findings, like an association between symptoms of gaming disorder and the speed of awareness acquisition^29^, unaware individuals rarely demonstrate PIT effects^10,17,28^. A way to overcome this problem is to omit the Pavlovian training phase of the PIT paradigm and to rely on naturally conditioned cues, e.g., alcohol- and tobacco-related pictures, in the transfer phase ^e.g.,26,30,31^. Although the design of these tasks deviates from the standard design of the PIT paradigm, they have been equally referred to as PIT paradigms ^e.g.,26,30,31^. This classification seems justified, as they measure the same mechanism, i.e., instrumental responding in the presence of conditioned cues, with the only difference that the conditioning is considered to have taken place outside the laboratory, in the natural environment of the participants. With regard to alcohol and tobacco, such naturally conditioned cues have proved to be effective in triggering PIT effects ^e.g.,26,30,31^. However, to the best of our knowledge, such a modified version of the PIT paradigm has not been implemented in the context of gaming and online shopping.

Regarding the cues presented in such short versions of the PIT paradigm, previous studies have used either pictures of the preferred drug (e.g., the preferred alcohol^26^), pictures of a popular drug (e.g., Becks beer^31^) or more prototypical pictures of the drug (e.g., plain cigarettes^30^). The first approach might have been guided by the assumption that cues of the preferred drug may be more effective, as has been observed in the context of craving and cue-reactivity, where effects were stronger with regard to preferred drinks^32^. Similar results have been found for behavioral addictions, with cues of preferred games^33^ or gambling modes^34^ eliciting higher craving and cue-reactivity. However, to the best of our knowledge, the role of cue preference has not been tested systematically with regard to the PIT effect.

Information is sparse not only regarding the role of individual preferences but also with regard to the reliability and stability of the PIT effect. Garbusow et al.^35^ investigated the reliability of drink- and money-related PIT effects over two halves of their PIT task. In contrast to the money-related PIT effect, which remained highly stable in detoxified patients with alcohol use disorder and the control group, the drink-related PIT effect, indicating response suppression in the presence of cues displaying alcoholic drinks, demonstrated high stability only in the patient group. Stability of a money-related PIT effect over a longer time period was studied by Chen et al.^36^, who observed weak to moderate stability over a time span of three years. Hence, while there is initial evidence of the reliability of the PIT effect, this information is limited to money- and drink-related PIT effects and to PIT paradigms that – depending on the trial – required individuals not only to perform but also to suppress responses to gain rewards. Furthermore, data on the stability over time spans shorter than three years is missing. This information is particularly relevant as researchers have started to test interventions – primarily approach-avoidance trainings – aimed at modifying the PIT effect^37,38^. To obtain a threshold against which to compare the effectiveness of such interventions, it is important to examine the stability of the PIT effect under similar conditions, but without the presence of an interposed intervention.

Against this background, the aim of the current study was twofold. First, this study intended to explore the effectiveness of a modified version of the PIT paradigm, in which naturally conditioned cues, i.e., gaming- and shopping-related pictures, instead of experimentally conditioned cues are presented during the transfer phase. This short PIT task may help to overcome the problem of individuals failing to acquire awareness of the stimulus-outcome associations in the Pavlovian training phase, a problem observed in previous PIT studies in the context of gaming and/or online shopping^14–17^. Additionally, it could allow a more economic assessment of gaming- and shopping-related PIT effects.

To gain insights into the effectiveness of both the gaming- and the shopping-related cues, this study involved a sample with game use and a sample with use of shopping websites. Regarding the PIT paradigm, we aimed to test the following preregistered hypotheses, with the target behavior being gaming in the gaming sample and shopping in the shopping sample.

### H1

Cues of the target behavior are expected to enhance responding for the reward related to the target behavior compared to the neutral stimulus (specific PIT effect), even after devaluation of this reward. Additionally, we explored whether responding for the reward related to the target behavior differed between cues displaying favorite games/shopping websites and cues displaying non-favorite games/shopping websites.

### H2

The magnitude of the specific PIT effect is positively associated with the symptom severity of the target behavior.

The second aim of this study was to examine the stability of the magnitude of the PIT effect, which can provide not only deeper insights into the nature of the PIT effect but also relevant information on the reliability of the PIT paradigm for studies aimed at modifying PIT effects. As recent studies tested approach-avoidance trainings as potential interventions, we decided to schedule a gaming-/shopping-related, non-training version of the approach-avoidance task between the two executions of the PIT paradigm, which we considered to be a comparable control condition. Given the sparsity of research, the retest reliability/stability of the PIT effect was explored without specific hypotheses.

## Methods

### Participants

For this study, we recruited 33 individuals who self-declared to use shopping websites at least occasionally in the last twelve months (shopping sample) and 33 individuals who self-declared to game at least occasionally in the last twelve months (gaming sample). Due to technical errors, two individuals in the shopping sample and one individual in the gaming sample had to be excluded, leaving final samples of 31 and 32 participants, respectively. The samples were recruited from the University of Bamberg and the Hannover Medical School by posts on social networks, mailing lists, flyers, and word-of-mouth recommendations. Inclusion criteria were a minimum age of 18 and at least good command of the German language. Engagement in the corresponding other behavior – online shopping for the gaming sample and gaming for the shopping sample – was assessed but was not a reason for exclusion. Participants were reimbursed with up to 30 euros for their participation or – if they were psychology students – could receive course credits.

The determination of our sample sizes was based on the recommendation of Hertzog^39^, who suggested 25 as lower threshold when testing instruments in a pilot study, but recommended samples sizes of 35 to 40 for investigating retest reliability. We hence aimed for a sample size of at least 25 and ideally 35 participants per sample; however, the latter target was not fully achieved.

### Procedure

The preregistered study consisted of a single experimental session, which lasted about two hours. The study procedure started with the first session of the PIT paradigm, followed by an approach-avoidance task with gaming- or shopping-related pictures and control stimuli. Subsequently, participants had a short break of 5–10 min, before performing the PIT paradigm for the second time. Finally, participants completed questionnaires assessing symptom severity of problematic gaming, symptom severity of problematic online shopping, and use-related experiences, and answered questions regarding sociodemographic information and their favorite games or favorite shopping websites. The study was conducted between July and October 2024.

### Measures

#### Pavlovian-to-instrumental transfer paradigm

**Fig. 1:**
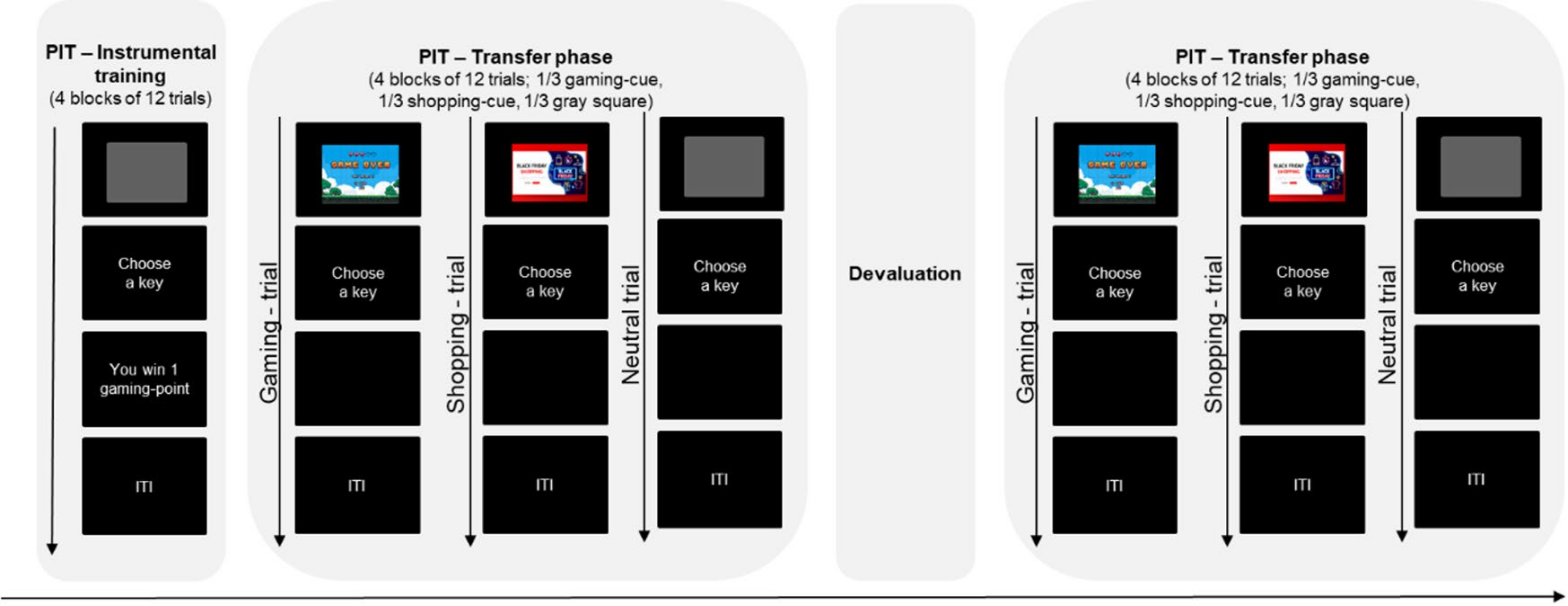
The short Pavlovian-to-instrumental-transfer paradigm. The figure displays the different phases of the PIT paradigm. The shopping and gaming pictures displayed are just sample pictures as the original cues used are subject to copyright. PIT = Pavlovian-to-instrumental-transfer; ITI = intertrial interval. © shopping & gaming picture: colourbox.

For the present study, we modified a PIT paradigm with gaming- and shopping-related stimuli and rewards, which we had used in our previous research^15,16^. Instead of associating abstract stimuli with gaming- and shopping-related pictures (i.e., screenshots of popular games or shopping websites) during the Pavlovian training, in the modified version, gaming- and shopping-related pictures were displayed in the transfer phase, which eliminated the need for a Pavlovian training phase. Hence, the modified version comprised only an instrumental training phase and a transfer phase (see Fig. 1), which were, apart from the use of gaming- and shopping-related pictures in the transfer phase, comparable to the PIT paradigm previously described in our research^15,16^. Specifically, the PIT paradigm still comprised a phase before and a phase after devaluation of rewards in the transfer phase (see below). The gaming and the shopping sample underwent the same PIT paradigm, i.e., both samples were presented with gaming- and shopping-related cues and rewards. The PIT paradigm was programmed in Presentation^®^ (Version 22.1, www.neurobs.com).

During the *instrumental training* of the PIT paradigm, individuals learned two different responses to earn gaming- and shopping-rewards, respectively. Each trial started with the presentation of a gray square (1600 × 900 pixels), which was displayed for 2.3 s and followed by the prompt “Please choose a key now! Press ‘S’ if you want to win shopping points and ‘G’ if you want to win gaming points.” To win a gaming or shopping point, participants had to press the respective key at least once during a 2-second response window. Consequently, participants learned that pressing the “G” key may result in earning gaming points and that pressing the “S” key may result in earning shopping points, however only 50% of the trials were rewarded. After the response window, a message appeared informing participants whether they had won a gaming point, a shopping point or nothing. The instrumental training consisted of four blocks with 12 trials each. To increase motivation, participants were told that they could exchange the gaming and shopping points won for gaming- and shopping-related vouchers, respectively. However, for ethical reasons, all participants received the same amount of compensation for study participation and were informed about this at the end of the study.

In the subsequent *transfer phase* of the PIT paradigm, responding was tested in the presence of gaming- and shopping-related stimuli. The transfer phase consisted of eight blocks with 12 trials each, with the devaluation scheduled after the first four blocks.

An instruction at the beginning of the transfer phase informed participants that either a picture or a gray square would precede each trial. However, the instruction did not imply that the stimuli predicted which response would be rewarded nor did it reveal that there was no contingency between the stimulus shown and the likelihood to earn rewards. Every trial started with the presentation of either a gaming-related picture, a shopping-related picture, or a neutral stimulus (i.e., gray square), with each stimulus being presented in one third of the trials per block. The stimuli were displayed with a size of 1600 × 900 pixel and stimulus presentation lasted 2.3 s. The shopping-related pictures consisted of screenshots from the eight most popular and best-selling shopping websites in Germany^40,41^, namely, Aboutyou, Amazon, Apple, Ikea, Lidl, Mediamarkt, Otto, Zalando. Similarly, for the gaming-related pictures, screenshots of gaming scenes of the eight most played, best-selling and/or most watched computer games^42–45^ were used (Call of Duty MWIII, Counterstrike 2, Grand Theft Auto V, Apex Legends, EA Sport FC 24, League of Legends, Minecraft, World of Warcraft). After stimulus presentation, the prompt “Please choose a key now! (‘S’ or ‘G’)” appeared, followed by a 2-second response window. As in the instrumental training, only 50% of the trials were rewarded, and reinforcement was independent of the stimulus shown. Hence, the stimulus displayed did not predict the likelihood to win gaming or shopping points. For example, if a gaming cue was presented and participants pressed the “G” key, they could either win a gaming point or win nothing. Similarly, if a gaming cue was presented and participants pressed the “S” key, they could either win a shopping point or win nothing. To prevent the learning of new stimulus-response associations^9,31^, responding was not followed by immediate feedback as in the instrumental training, but participants received an overview of the number of gaming and shopping points won only at the end of the task. This approach ensured that participants’ responses reflected their pre-learned associations between the stimuli and the expected rewards rather than associations formed during the transfer phase.

Stimulus-congruent responses in the transfer phase could be guided by the (incorrect) assumption that these response are more likely to be rewarded (goal-directed account^9^) or reflect habit-like responses towards these stimuli as result of an associative S-O-R chain^9,19–22^. To identify the underlying mechanism, the gaming- or shopping-related reward was devalued after four blocks of the transfer phase. The devaluation consisted of a text informing participants that they had reached the maximum amount of gaming/shopping points in this part of the experiment and that they would not be able to earn more gaming/shopping points. For participants in the gaming sample, the gaming-related reward was devalued, and for participants in the shopping sample, the shopping-related reward was devalued. The transfer phase was therefore divided into two phases, with four blocks each: before and after devaluation of the specific reward. After devaluation, responses for the devalued reward did not earn any rewards, i.e., no points were added to the winnings displayed at the end of the task.

Participants performed the same PIT procedure in the first and second session. Hence, outcomes were devalued twice, once in the transfer phase of the first PIT session and once in the transfer phase of the second PIT session.

The following outcome measures were calculated for the PIT paradigm: As indicators of the response behavior, the aggregated choice of the gaming-related response (percentage of trials in which the gaming-related key was pressed compared to all trials) and the aggregated choice of the shopping-related response (percentage of trials in which the shopping-related key was pressed compared to all trials) were calculated. The magnitude of the gaming PIT effect (for both before and after devaluation) was computed as difference between the choice of the gaming-related response (“G” presses) in gaming trials and the choice of the gaming-related response (“G” presses) in trials, in which the gray square was displayed. Consequently, the magnitude of the shopping PIT effect expressed the difference between the choice of the shopping-related response (“S” presses) in shopping trials and the choice of the shopping-related response (“S” presses) in neutral trials. The magnitude of the PIT effect was computed separately for the blocks before and after devaluation. While the magnitude of the PIT effect before devaluation can be seen as an indicator for cue-elicited responding in general, the magnitude of the PIT effect after devaluation may be interpreted as indicator of habit-like responding towards reward-related cues. In addition to the PIT effects before and after devaluation, a selectivity index as described by Holmes et al.^46^ was computed. The selectivity index represents the difference in responding towards a stimulus associated with the same and a stimulus associated with a different outcome. Consistently, in our sample, the selectivity index was computed as difference between responding towards the gaming stimuli and responding towards the shopping stimuli.

#### Approach-avoidance task

We used a non-training version of the Approach-Avoidance Task (AAT)^47^ with gaming- or shopping-related pictures (depending on the sample) and neutral pictures. The gaming pictures (used in the gaming sample) showed log-in or start screens of video games, while the shopping pictures (used in the shopping sample) depicted log-in pages of shopping websites. The neutral pictures showed stationary objects (e.g., pencil, scissors,…). In each block, one stimulus category had to be pulled towards the participant, and the other category had to be pushed away by moving a joystick. Pulling the joystick towards oneself increased the size of the pictures, while pushing it away decreased the size of the pictures. Which stimulus category was designated to be pulled towards oneself or pushed away was counterbalanced across blocks. Hence, the number of trials in which the gaming/shopping stimulus had to be approached and the number of trials in which the gaming/shopping stimulus had to be avoided were equal. The task consisted of four blocks and each block comprised 40 gaming- /shopping-related pictures and 40 control pictures.

#### Questionnaires

##### Assessment of criteria for specific internet-use disorders

Symptom severity of problematic online shopping and gaming was assessed with the Assessment of Criteria for Specific Internet-use Disorders (ACSID-11)^48^, a screening instrument which is based on the ICD-11 criteria for gaming disorder^49^. Symptoms are rated regarding how frequently (0 = *never*, 1 = *rarely*, 2 = *sometimes*, 3 = *often*) and how intensively (0 = *not at all intense*, 1 = *rather not intense*, 2 = *rather intense*, 3 = *intense*) they are experienced. For the present study, a mean score of the frequency response scale^48^ was used.

##### Ten-item internet gaming disorder test

Symptoms of gaming disorder were additionally assessed with the Ten-Item Internet Gaming Disorder Test (IGDT-10)^50^, which covers the DSM-5 criteria for gaming disorder. Except for criterion nine, which is covered by two items, each criterion is measured by one item. Items are rated on a 3-point Likert scale labeled with 0 = *never*, 1 = *sometimes*, 2 = *often*. The criterion is considered met, if the corresponding item – or in case of criterion nine, one of the two items – is answered with *often.* A sum score, representing the number of fulfilled criteria, was calculated. Pathological gaming behavior is assumed if participants score 5 or higher^50^.

##### Pathological buying screener

Symptom severity of problematic shopping was further assessed with the Pathological Buying Screener (PBS)^51^, which refers not only to online shopping but to shopping in general. The 13 items of the scale are assessed on a 5-point Likert scale ranging from 1 (*never*) to 5 (*very frequently*). For the present study a total score, derived by summarizing all items, was used. Pathological buying-shopping is indicated from a value of 29^52^.

##### Experience of gratification scale and experience of compensation scale

To measure experience of gratification and compensation with regard to online shopping and gaming, we used the Experience of Gratification Scale (EGS)^53^ and the Experience of Compensation Scale (ECS)^53^. Each scale comprises six items, which are rated on a 5-point Likert scale from 0 = *never* to 4 = *very often.* Total mean scores were computed for both scales.

##### Identification of favorite games and favorite shopping websites

The gaming sample was presented a list with the games included in the PIT paradigm. Participants should indicate which, if any, of the games were their favorite one(s). Similarly, the shopping sample was presented a list with the shopping websites included in the PIT paradigm and was asked to indicate which, if any, of the shopping websites were their favorite one(s).

### Statistical analysis

As different outcomes were devalued in the samples (shopping was devalued in the shopping sample and gaming in the gaming sample), all analyses were conducted separately for the two samples.

Since inspection of the data revealed non-normal distributions, we conducted multilevel logistic regressions (trials nested in participants) to analyze the effects of stimulus, devaluation, and time on response choice in the transfer phase. The repeated-measures ANOVAs described in the preregistration are reported as sensitivity analyses in the supplementary material. Analyses on block effects can also be found in the supplementary material. Response choice in each trial of the transfer phase was used as outcome measure in the multilevel logistic regressions, with choice of the gaming-related response being the relevant outcome in the gaming sample and choice of the shopping-related response being the relevant outcome in the shopping sample. While choice of the relevant response was coded as 1, choice of the respective other response and non-responding were both coded as 0. We decided to treat non-responding and choosing the alternative response equally, as both present a choice against the response of interest (gaming in the gaming sample and shopping in the shopping sample) and both may be indicative of goal-directed behavior after the devaluation. Stimulus (dummy coded with neutral stimulus as reference category), devaluation (before/after, coded as 0/1), and time (1. PIT session/2. PIT session, coded as 0/1) and their interactions were modelled as fixed effects. Additionally, we included random intercepts for participants and random slopes for stimulus, devaluation, and time. In both samples, intraclass correlations (ICC) indicated that a greater proportion of the variance in response choice could be attributed to within- than between-subject differences (gaming sample: ICC_gaming choice_= 0.09; shopping sample: ICC_shopping choice_= 0.31).

Additionally, we computed multilevel logistic regressions to test whether the response choice during the transfer phase differed when the stimulus shown depicted a favorite game/shopping website compared to a non-favorite game/shopping website. These analyses were conducted only for the first session of the PIT paradigm. Since some individuals indicated none of the games/shopping websites included in the PIT paradigm as their favorites, this analysis was conducted in reduced samples (gaming: *n* = 23; shopping: *n* = 26). Preference (favorite/non-favorite, coded as 1/0) and devaluation (before/after, coded as 0/1) were included as fixed effects. The models were computed with random intercepts only, since the model with favorite stimulus as random slope did not demonstrate a better fit in the gaming sample (LR χ² (2) = 0.37, *p* = 0.83) and yielded singular fit in the shopping sample. As this analysis was not included in our preregistration, it presents an exploratory analysis. (In line with the main research question, we also report results of repeated-measures ANOVAs as sensitivity analyses in the supplementary material).

The multilevel logistic regressions were calculated in R (version 4.3.3)^54^ with the lme4 package^55^, using the optimizer bobyqa, and the results were visualized with the ggplot2 package^56^.

To test associations between the magnitude of the gaming/shopping PIT effect at t1 and symptom severity, Spearman correlations were computed. The stability/retest reliability of the PIT effect was explored by calculating Spearman correlations between the magnitude of the PIT effect at t1 and the magnitude of the PIT effect at t2. Spearman correlations were chosen due to the non-normal distribution of the data. In addition to *p* values, 95% confidence intervals for Spearman correlations with Fieller et al.’s standard error^57,58^ are reported (one-tailed confidence intervals for the directional hypothesis on associations with symptom severity and two-tailed confidence intervals for the retest reliability). In addition to the Spearman correlation as measure of relative consistency between the two time points, ICC estimates and their 95% confidence intervals were computed as measure of absolute consistency. The calculation of the ICCs was based on a single measurement, absolute-agreement, 2-way mixed-effects model^59^. ICC values less than 0.50 are considered to reflect poor reliability, values between 0.50 and 0.75 moderate reliability, values between 0.75 and 0.90 good reliability, and values above 0.90 excellent reliability^59^.

The retest reliability and associations with symptom severity were also explored for the selectivity index of the PIT paradigm. However, as the primary focus of our paper was on the PIT effect, results concerning the selectivity index are reported in the supplementary material (Table A1 and A2). Spearman correlations, ICCs and descriptive statistics were calculated with IBM SPSS Statistics for Windows, version 29 (IBM Corp., Armonk, N.Y., USA). For all analyses, the significance level was set at 0.05.

### Openness and transparency

The study was preregistered at OSF: https://osf.io/wtzxm/?view_only=55854afcf18c4ce0841b0e1111221af7.

## Results

### Sample characteristics

Table 1 displays the sample characteristics of both samples. The gender distribution was biased toward males in the gaming sample, and towards females in the shopping sample. Both samples displayed higher symptom severity for their respective target behavior, however, mean symptom severity was below the cutoffs suggested for pathological behavior.

**Table 1:** Description of samples.

Sample characteristics	Gaming sample (*N* = 32)	Shopping sample (*N* = 31)	Cronbach’s α
Gender, *n* (%)			
Female	8 (25.0)	23 (74.2)	
Non-binary		1 (3.2)	
Male	24 (75.0)	7 (22.6)	
Age (years)	24.8 (3.6)	28.8 (8.9)	
Education (school years)	12.8 (0.6)	12.8 (0.8)	
Use time workday gaming/shopping (minutes)	105.8 (86.4)	31.6 (53.0)	
Use time weekend gaming/shopping (minutes)	239.1 (188.9)	67.6 (71.1)	
Symptom severity gaming (ACSID-11 gaming) ^a^	0.5 (0.5)	0.2 (0.3)	0.85
Symptom severity shopping (ACSID-11 shopping) ^b^	0.2 (0.3)	0.4 (0.6)	0.92
Symptom severity gaming (IGDT-10) ^a^	0.8 (1.4)	0.1 (0.5)	0.83
Symptom severity shopping (PBS)	20.0 (7.1)	25.2 (10.7)	0.94
Experienced gratification due to gaming/shopping (EGS)	2.5 (0.7)	1.5 (0.7)	0.76/ 0.80
Experienced compensation due to gaming/shopping (ECS)	1.6 (1.1)	0.9 (0.8)	0.92/ 0.88

All values are displayed as means and standard deviations in brackets if not otherwise specified. ACSID-11 = Assessment of Criteria for Specific Internet-Use Disorders; IGDT-10 = Ten-Item Internet Gaming Disorder Test; PBS = Pathological Buying Screener; EGS = Experience of Gratification Scale; ECS = Experience of Compensation Scale.

^a^The IGDT-10 and the ACSID-11 gaming were only presented to individuals who indicated to have engaged in gaming at least occasionally in the last 12 months. In the shopping sample, this applied to only 14 participants.

^b^The ACSID-11 shopping was only presented to individuals who indicated to have engaged in online shopping at least occasionally in the last 12 months. In the gaming sample, this applied to 30 participants.

### Responding in the presence of gaming- and shopping-related stimuli

**Fig. 2:**
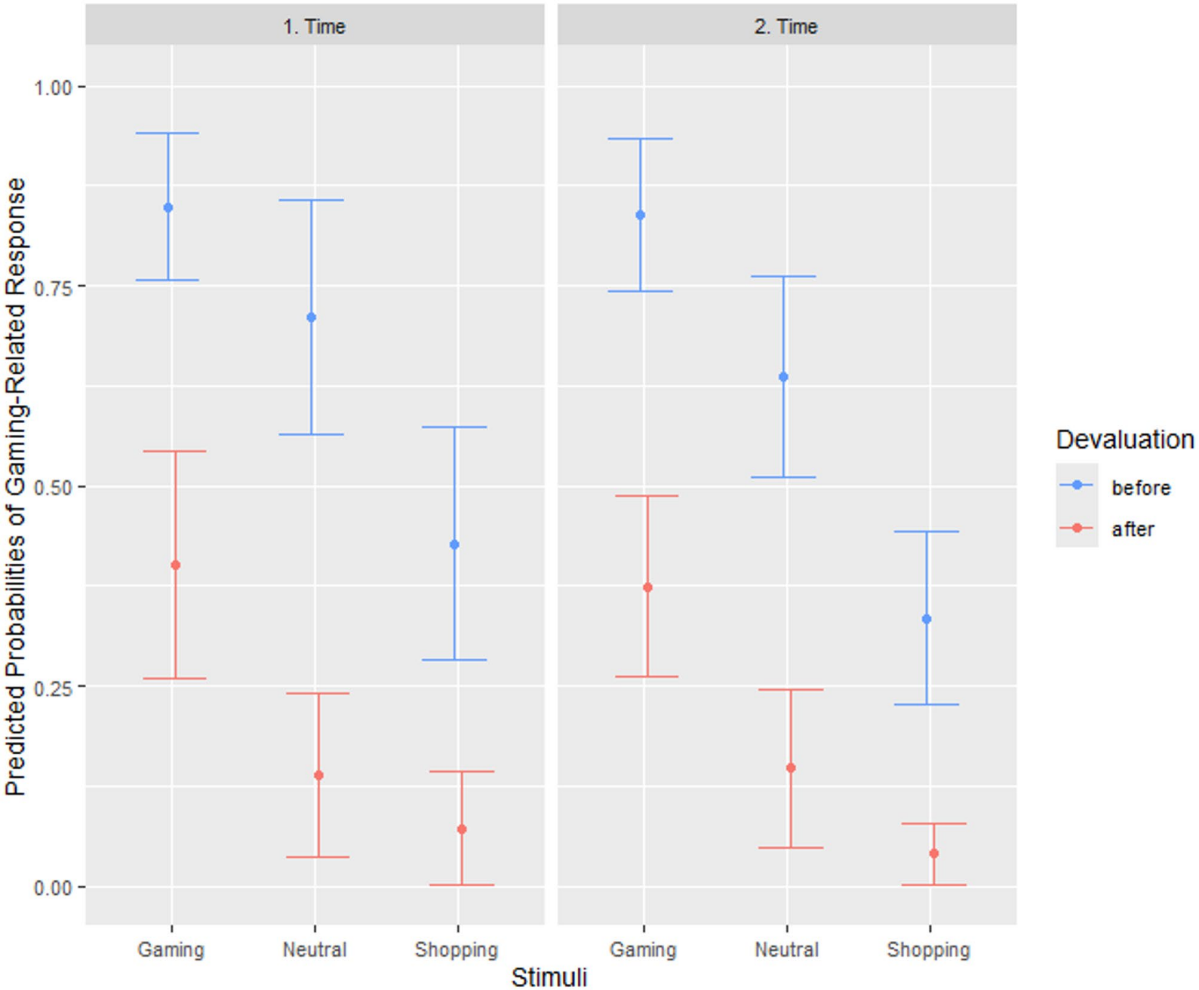
Predicted probabilities of the gaming-related response – effect of stimulus, devaluation, and time. Predicted probabilities and 95% confidence intervals of the gaming-related response after presentation of the gaming-related stimuli, the neutral stimulus (gray square), and the shopping-related stimuli before and after devaluation at the first and second session of the PIT paradigm. *N* = 32.

In the gaming sample, analyses of response choice revealed a main effect of the gaming- and shopping-related stimuli (see Table 2). While presentation of the gaming-related stimuli significantly increased the likelihood of the gaming-related response compared to the neutral stimulus, presentation of the shopping-related stimuli decreased the likelihood of the gaming-related response. Overall, devaluation decreased the likelihood of the gaming-related response, however the decrease was smaller in the presence of the gaming-related stimuli, as indicated by a significant gaming stimulus by devaluation interaction. Even after devaluation, the likelihood of the gaming-related response was highest after the presentation of the gaming-related stimuli (see Fig. 2). Thus, a gaming PIT effect could still be observed. Finally, a significant time by devaluation interaction emerged, which indicated a smaller devaluation effect in the second session of the PIT paradigm.

**Fig. 3:**
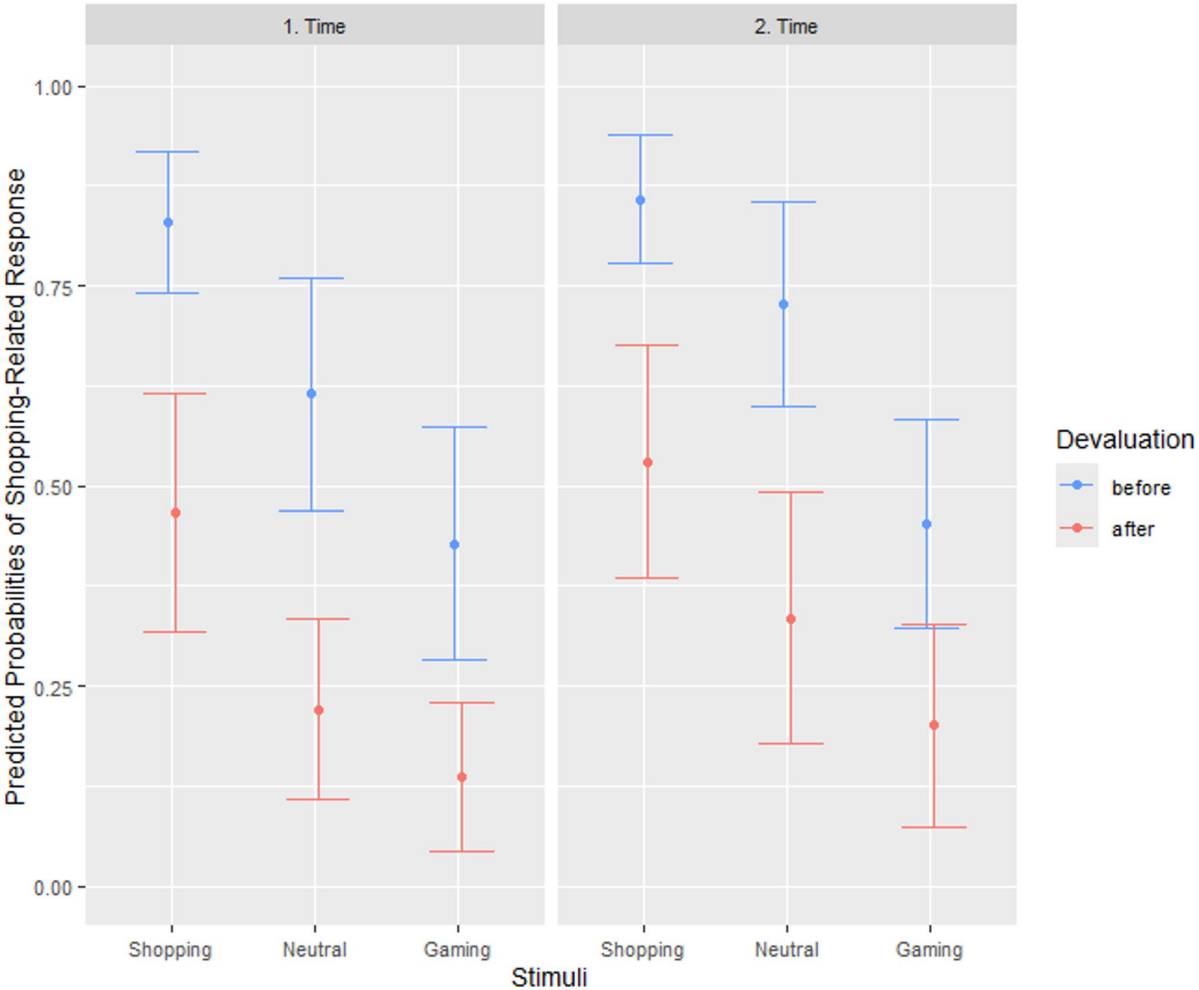
Predicted probabilities of the shopping-related response – effect of stimulus, devaluation, and time. Predicted probabilities and 95% confidence intervals of the shopping-related response after presentation of the shopping-related stimuli, the neutral stimulus (gray square), and the gaming-related stimuli before and after devaluation at the first and second session of the PIT paradigm. *N* = 31.

Significant effects of stimulus and devaluation were also observed in the shopping sample (see Table 2). Individuals adapted their choice of the shopping-related response to the stimulus presented, with the shopping-related stimuli increasing the likelihood of the shopping-related response compared to the neutral stimulus and the gaming-related stimuli decreasing the likelihood of the shopping-related response. The likelihood of the shopping-related response was decreased after devaluation, however, the shopping PIT effect, i.e., increased likelihood to choose the shopping-related response after presentation of the shopping-related stimuli, was still visible (see Fig. 3). A significant main effect of time indicated that the likelihood of the shopping-related response was increased in the second session of the PIT paradigm. This time-dependent increase was smaller after presentation of the gaming-related stimuli, as indicated by a gaming stimulus by time interaction.

Results of the repeated-measures ANOVAs using aggregated values (see supplemental material) replicated the main effects of stimulus and devaluation in both samples, the main effect of time in the shopping sample and the stimulus by devaluation interaction in the gaming sample. A closer look at the distribution of the individual aggregated data points (see Figure A1a in the supplementary material) revealed that the devaluation effect differed considerably between participants in the gaming sample, especially after the presentation of the gaming stimuli. While some individuals successfully refrained from choosing the gaming-related response and others at least reduced their choice, a third group kept choosing the gaming-related response whenever the gaming stimuli were presented. A comparable pattern with regard to the shopping-related response in the presence of the shopping-related stimuli was observed in the shopping sample (see Figure A1b in the supplementary material).

**Table 2:** Effect of stimulus, devaluation, and time on response choice.

	Gaming sample: Choice of gaming-related response				Shopping sample: Choice of shopping-related response			
	*B*	OR	95% CI	*p*	*B*	OR	95% CI	*p*
Gaming-related stimulus^a^	**1.669**	**5.307**	**[2.212; 12.733]**	**< 0.001**	**-2.028**	**0.132**	**[0.039; 0.446]**	**0.001**
Shopping-related stimulus^b^	**-2.206**	**0.110**	**[0.046; 0.266]**	**< 0.001**	**1.932**	**6.904**	**[2.310; 20.631]**	**0.001**
Devaluation^c^	**-6.010**	**0.002**	**[0.001; 0.012]**	**< 0.001**	**-3.834**	**0.022**	**[0.004; 0.123]**	**< 0.001**
Time^d^	-0.716	0.489	[0.194; 1.230]	0.129	**1.236**	**3.441**	**[1.348; 8.784]**	**0.010**
Gaming-related stimulus*devaluation	**0.743**	**2.102**	**[1.072; 4.121]**	**0.031**	-0.373	0.689	[0.347; 1.367]	0.286
Shopping-related stimulus*devaluation	0.484	1.623	[0.792; 3.322]	0.186	0.210	1.234	[0.652; 2.337]	0.518
Gaming-related stimulus*time	0.447	1.564	[0.877; 2.789]	0.129	**-0.771**	**0.463**	**[0.248; 0.862]**	**0.015**
Shopping-related stimulus*time	-0.326	0.722	[0.425; 1.227]	0.228	-0.369	0.691	[0.359; 1.330]	0.269
Time* devaluation	**0.975**	**2.651**	**[1.461; 4.812]**	**0.001**	-0.216	0.806	[0.441; 1.472]	0.482
Gaming-related stimulus*devaluation*time	-0.828	0.437	[0.180; 1.057]	0.066	0.788	2.200	[0.881; 5.494]	0.091
Shopping-related stimulus*devaluation*time	-0.326	0.722	[0.273; 1.907]	0.511	-0.071	0.931	[0.380; 2.280]	0.876

Results of multilevel logistic regressions of stimulus, devaluation, and time on choice of the gaming-/shopping-related response. The models were estimated with random intercepts and random slopes for stimulus, devaluation, and time. Confidence intervals were estimated with the Wald’s method. Significant effects are highlighted in bold. Gaming sample: *N*_*individuals*_ = 32; *N*_*observations*_ = 6144; Shopping sample: *N*_*individuals*_ = 31; *N*_*observations*_ = 5952. OR = odds ratio, CI = confidence interval.

^a^ 0 = shopping-related/neutral stimulus, 1 = gaming-related stimulus. ^b^ 0 = gaming-related/neutral stimulus, 1 = shopping-related stimulus. ^c^ 0 = before devaluation, 1 = after devaluation. ^d^ 0 = first session of the PIT paradigm, 1 = second session of PIT paradigm.

### Responding in the presence of stimuli of favorite games or shopping websites

**Fig. 4:**
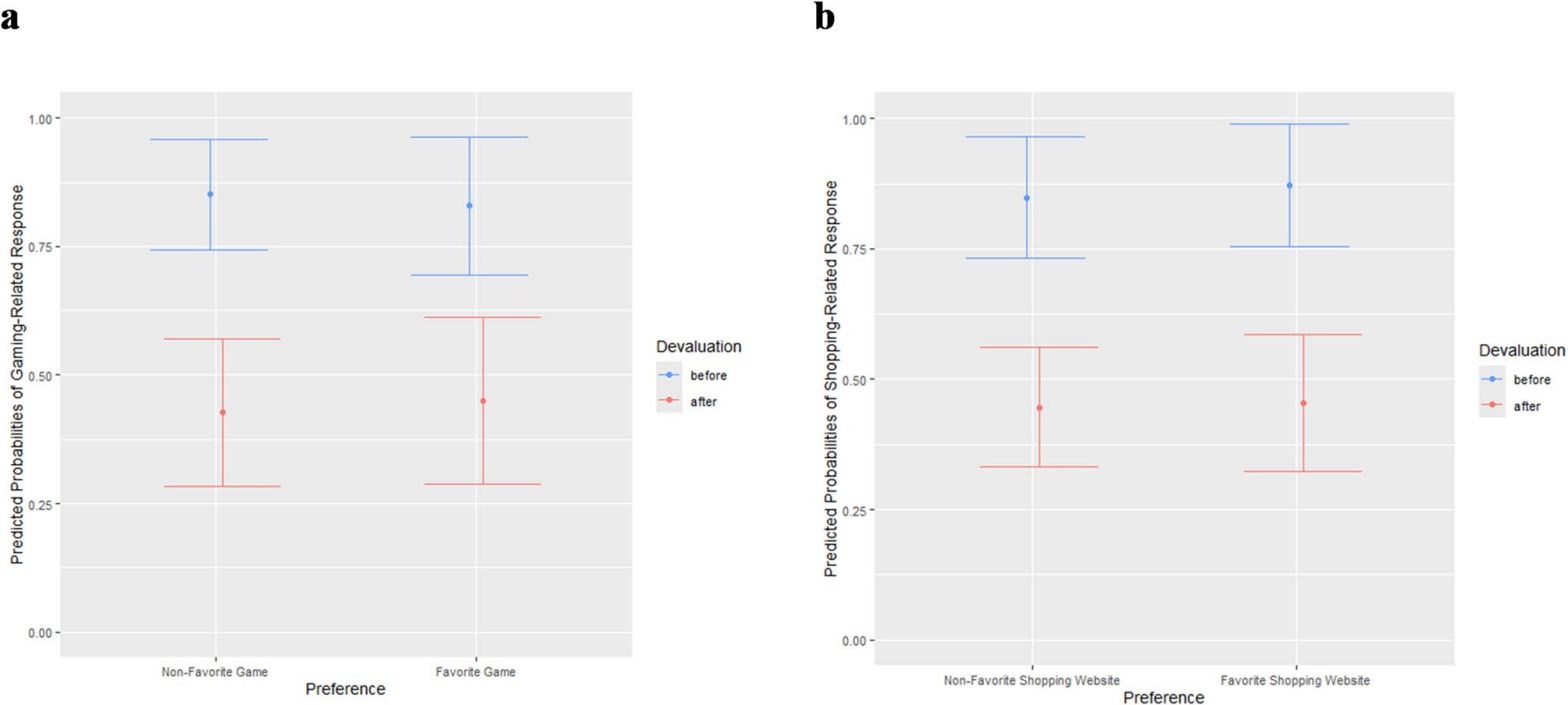
Predicted probabilities of the gaming-/shopping-related response – effect of favorite game/shopping website. (**a**) Predicted probabilities and 95% confidence intervals of the gaming-related response after presentation of stimuli depicting favorite games compared to stimuli depicting non-favorite games before and after devaluation at the first PIT session. *n* = 23. (**b**) Predicted probabilities and 95% confidence intervals of choice of shopping-related response after presentation of stimuli depicting favorite shopping websites compared to stimuli depicting non-favorite shopping websites before and after devaluation at the first PIT session. *n* = 26.

Participants in the gaming sample displayed a similar preference for the gaming-related response after presentation of stimuli depicting their favorite game(s) and stimuli depicting non-favorite game(s) (see Table 3; Fig. 4a). Furthermore, the devaluation effect did not differ between stimuli depicting favorite games and stimuli depicting non-favorite games, as indicated by a non-significant preference by devaluation interaction. After devaluation, the likelihood to choose the gaming-related response was reduced, however only to some degree.

Similarly, in the shopping sample, the likelihood of the shopping-related response did not differ between stimuli displaying favorite shopping website(s) and stimuli depicting non-favorite shopping websites (see Table 3; Fig. 4b). The effect of the devaluation was comparable across both types of stimuli, as pointed out by a non-significant preference by devaluation interaction. Hence, participants were less likely to choose the shopping-related response after devaluation regardless of whether a stimulus depicted a favorite or non-favorite shopping website.

Results of the repeated-measures ANOVAs using aggregated values (see supplementary material) replicated the results of the multilevel logistic regressions, indicating no significant main effect of preference nor a significant preference by devaluation interaction.

**Table 3:** Effect of favorite game/shopping website on response choice.

	Gaming sample: Choice of gaming-related response				Shopping sample: Choice of shopping-related response			
	*B*	OR	95% CI	*p*	*B*	OR	95% CI	*p*
Favorite game/shop^a^	-0.283	0.753	[0.302; 1.878]	0.543	0.243	1.275	[0.585; 2.780]	0.541
Devaluation^b^	**-3.959**	**0.019**	**[0.009; 0.039]**	**< 0.001**	**-3.188**	**0.041**	**[0.024; 0.071]**	**< 0.001**
Favorite game/shop*devaluation	0.509	1.664	[0.502; 5.510]	0.405	-0.166	0.847	[0.296; 2.429]	0.758

Results of multilevel logistic regressions of favorite game/shopping website and devaluation on choice of gaming-/shopping-related response for the first session of the PIT paradigm. The models were estimated with random intercepts. Confidence intervals were estimated with the Wald’s method. Significant effects are highlighted in bold. Gaming sample: *n*_*individuals*_ = 23; *n*_*observations*_ = 736; Shopping sample: *n*_*individuals*_ = 26; *n*_*observations*_ = 832. OR = odds ratio, CI = confidence interval.

^a^ 0 = non-favorite game/shopping website, 1 = favorite game/shopping website. ^b^ 0 = before devaluation, 1 = after devaluation.

### Associations between the magnitude of the PIT effect and symptom severity

The magnitude of the gaming PIT effect before devaluation was not significantly correlated with symptom severity as measured with the IGDT-10 (*r*_*s*_ = 0.25, one-tailed 95% CI [-0.06, 1], *p* = 0.08), nor with symptom severity as assessed with the ACSID-11 (*r*_*s*_ = 0.18, one-tailed 95% CI [-0.13, 1], *p* = 0.16). Regarding the magnitude of the gaming PIT effect after devaluation, it was barely correlated with the ACSID-11 (*r*_*s*_ = 0.02, one-tailed 95% CI [-0.29, 1], *p* = 0.46), while its association with the IGDT-10 approached significance (*r*_*s*_ = 0.29, one-tailed 95% CI [-0.01, 1], *p* = 0.05).

Regarding the magnitude of the shopping PIT effect before devaluation, no significant association with symptom severity of problematic shopping as measured with the ACSID-11 (*r*_*s*_ = 0.15, one-tailed 95% CI [-0.17, 1], *p* = 0.21) or the PBS (*r*_*s*_ = 0.04, one-tailed 95% CI [-0.28, 1], *p* = 0.42) emerged. The same applied to the shopping PIT effect after devaluation (correlation with ACSID-11: *r*_*s*_ = 0.15, one-tailed 95% CI [-0.17, 1], *p* = 0.22; correlation with PBS: *r*_*s*_ = − 0.12, one-tailed 95% CI [-0.41, 1], *p* = 0.26).

### Stability/retest reliability of the magnitude of the PIT effect

**Fig. 5:**
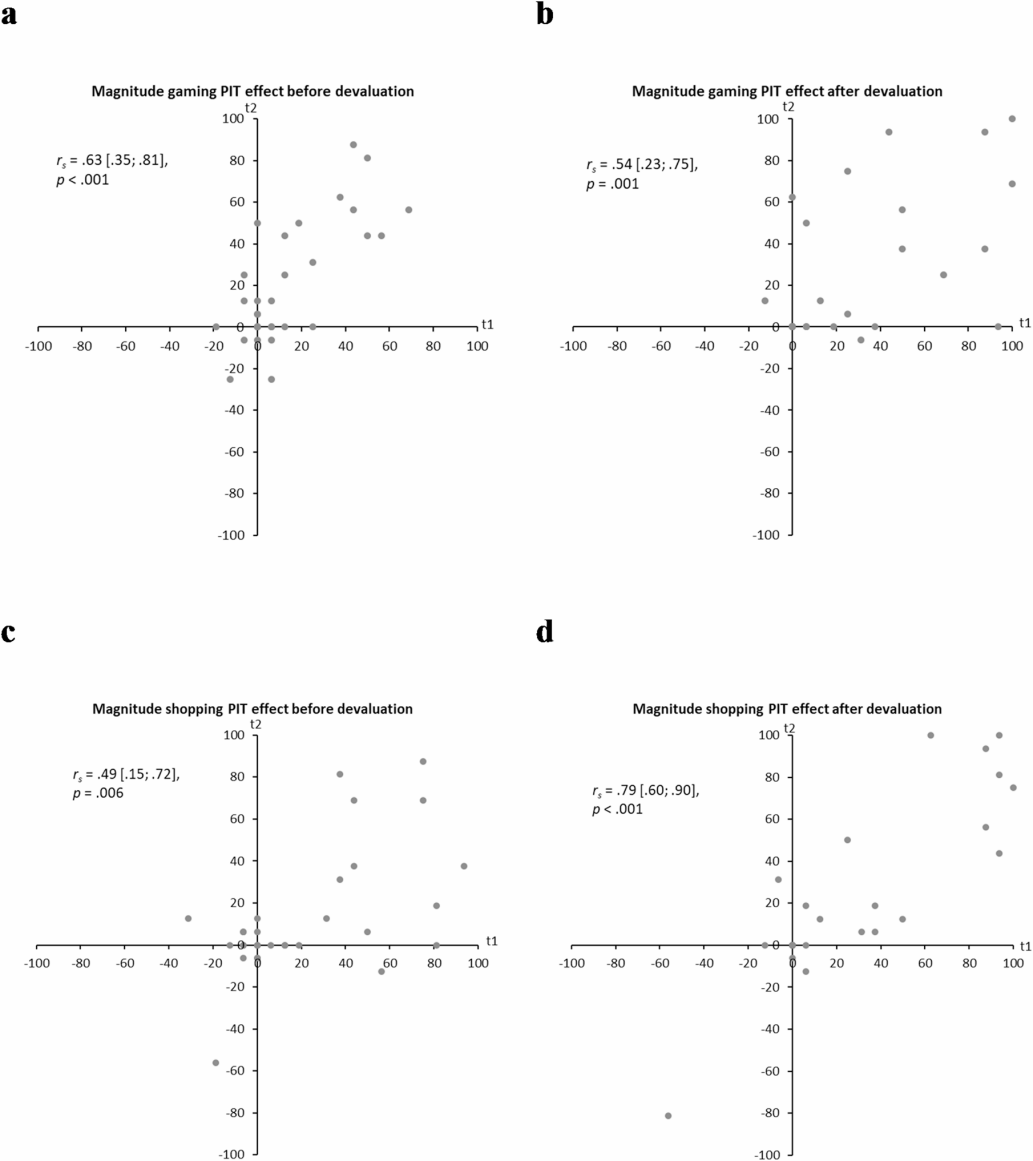
Stability of the magnitude of the PIT effect between t1 and t2. (**a**) Correlation of the magnitude of the gaming PIT effect before devaluation between t1 and t2; *N* = 32. (**b**) Correlation of the magnitude of the gaming PIT effect after devaluation between t1 and t2; *N* = 32. (**c**) Correlation of the magnitude of the shopping PIT effect before devaluation between t1 and t2; *N* = 31. (**d**) Correlation of the magnitude of the shopping PIT effect after devaluation between t1 and t2; *N* = 31. Because of the non-normal distribution of the data, Spearman correlations were computed. The values presented in square brackets refer to the 95% confidence intervals.

For the gaming sample, the magnitude of the gaming PIT effect both before and after devaluation displayed significant strong correlations between both time points (see Fig. 5a,b). Similarly, the magnitude of the shopping PIT effect both before and after devaluation showed significant and strong associations between both time points (see Fig. 5c,d). However, for both gaming PIT effects and the shopping PIT effect before devaluation, the 95% confidence intervals were large.

The additionally estimated ICCs indicated moderate retest reliability for the gaming PIT effect before devaluation (ICC = 0.71, 95% CI [0.48; 0.85], *p* < 0.001) and after devaluation (ICC = 0.61, 95% CI [0.33; 0.79], *p* < 0.001). The same applied to the shopping PIT effect before devaluation (ICC = 0.56, 95% CI [0.27; 0.76], *p* < 0.001), while the shopping PIT effect after devaluation displayed good retest reliability (ICC = 0.87, 95% CI [0.75; 0.94], *p* < 0.001). When considering the 95% confidence intervals of the ICCs as suggested by Koo and Li^59^, retest reliabilities ranged from poor to good for the gaming PIT effects and the shopping PIT effect before devaluation, and from moderate to excellent for the shopping PIT effect after devaluation.

## Discussion

The aim of our pilot study was to test a short version of the Pavlovian-to-Instrumental Transfer Paradigm with gaming- and shopping-related stimuli and rewards. Additionally, we investigated the retest reliability/stability of the PIT effect over two repeated sessions of the PIT paradigm.

Our modified PIT paradigm, which worked with naturally instead of experimentally conditioned cues, hence making the Pavlovian training phase obsolete, proved to be effective in triggering cue-elicited responding. While pictures of gaming scenes enhanced responding for the gaming-related reward, pictures of shopping websites increased responding for the shopping-related reward. This finding parallels results from tobacco- and alcohol-related PIT paradigms, which also indicated the effectiveness of naturally conditioned cues in triggering responding for the congruent reward ^e.g.,26,30,31^.

In line with previous studies, we combined our PIT paradigm with a devaluation procedure to establish whether the cue-elicited responding is more in line with habitual or goal-directed behavior. The results were comparable to those of our previous studies in which we utilized a longer version of the PIT paradigm^15,16^. Even after devaluation, the gaming- and shopping-related stimuli triggered responding for the congruent rewards, albeit to a smaller degree. This PIT effect after devaluation is in line with the habitual account of PIT, according to which the PIT effect is the result of a S-O-R chain^9,19–22^, which encodes only the sensory properties but not the current motivational value of the outcome^9,21^.

In light of our findings, our short modified version of the PIT paradigm may present an attractive alternative to the classical PIT paradigms in the context of gaming and online shopping. Omitting the Pavlovian training phase not only creates a more economic version of the PIT paradigm but also solves the issue of individuals failing to learn the Pavlovian contingencies. The latter has posed a problem in previous studies, as individuals unaware of the stimulus-outcome contingencies were unlikely to show a PIT effect^10,17,28^. Moreover, our modified PIT paradigm could be considered more ecologically valid, as it incorporates cues that individuals commonly encounter in their daily lives.

Remarkably, responding for gaming- or shopping-related rewards did not differ between cues of favorite and non-favorite games and shopping websites (neither before nor after devaluation). Hence, cue-elicited responding appears to generalize to cues associated with the behavior in general, independently of individuals’ preferences. In this regard, cue-elicited responding seems to differ from craving and cue-reactivity, which have been found to be more pronounced towards preferred drinks^32^, games^33^ or gambling mode^34^. Thus, even if cues of non-favorite content may elicit lower levels of craving, they may still trigger engagement in the addictive behavior. From a methodological perspective, the observed generalization effect implies that individualizing cues for the PIT paradigm may not be necessary – at least regarding gaming- and shopping-related cues. However, as this analysis was based only on a subsample of participants, which further reduced the sample size, the finding must be considered with caution until replicated in a larger sample.

Concerning the second aim of our study, i.e., exploring the stability/retest reliability of the PIT effect (both before and after devaluation), reliability estimates (Spearman correlations and ICCs) indicated, on average, moderate stability across the two time points, which were separated by a 30–40 min interval, in which participants performed the AAT followed by a short break. The size of the Spearman correlations was comparable to those observed by Garbusow et al.^35^, who investigated the split-half reliability of the PIT paradigm, and higher than those reported for a time interval of three years^36^. However, most of the confidence intervals were large, and hence the results must be interpreted with the necessary caution until supported in prospective studies. If the findings of this proof-of-concept study can be replicated in larger and clinical studies and across longer retest intervals, our PIT task may prove helpful for studies which aim to test interventions to modify the PIT effect and intend to use a pre-post design with repeated administrations of the PIT paradigm. For the selectivity index (both before and after devaluation), we observed good retest reliability across both time points suggesting that this may also be a valid indicator for cue-elicited responding for studies interested in contrasting two reward-related responses.

While the magnitude of the PIT effect (both before and after devaluation) was found to be moderately stable on the individual level, individuals in the shopping sample increased their choice of the shopping-related response from the first to the second time point. Familiarity with the task may be one possible explanation for this result. Alternatively, the feedback provided after the first session on the gaming and shopping points won may have served as additional reinforcer, motivating participants in the shopping sample to more frequently choose the shopping-related response in the second session. A comparable rationale might be behind the smaller devaluation effect in the second session of the PIT paradigm observed in the gaming sample. The winnings provided after the first session usually included gaming and shopping points, as participants received the combined sum of points earned before and after devaluation. Earning both shopping and gaming points might have obscured the fact that the devaluation eliminated the availability of the gaming points, hence limiting the effectiveness of the devaluation in the second PIT session. On the other hand, increased choice of the gaming/shopping response in the second session could have been triggered by the confrontation with gaming-/ shopping-related stimuli during the AAT, which might have enhanced craving or the desire to engage in gaming or shopping.

In the gaming sample, the magnitude of the gaming PIT effect after devaluation displayed a medium-sized correlation with symptom severity of problematic gaming, as measured with the IGDT-10, which only slightly missed significance. Although the large confidence interval questions the robustness of this result, it is in line with previous studies reporting an association between the gaming PIT effect^14^, and especially the gaming PIT effect after devaluation^15^, and symptoms of gaming disorder. In contrast, the shopping PIT effect (both before and after devaluation) was not associated with symptom severity in the shopping sample, which replicates previous results^17^. Potentially, associations between the shopping PIT effect and symptom severity may be more complex, as suggested by a recent study^16^. In this study, symptom severity predicted the shopping PIT effect (after devaluation) only in interaction with responsivity to acute stress. Differential results with regard to gaming and shopping were also observed for the selectivity index: Only the association between the selectivity index before devaluation and symptom severity of problematic gaming (measured with the IGDT-10), but none of the associations with symptom severity of problematic shopping, approached statistical significance.

Inspection of the individual response choices (aggregated across trials, see Figure A1 in the supplementary material), revealed that the devaluation effect differed considerably between participants, especially after the presentation of the stimuli signaling the devalued outcome. While some individuals successfully stopped responding for the devalued reward, others did hardly modify their behavior. In light of the mostly non-significant associations with symptom severity, this variability may be due to other factors. It may be promising to explore which inter-individual differences can account for this effect.

### Strengths, limitations, and suggestions for future studies

To the best of our knowledge, this study is the first to implement a short version of the PIT paradigm, as it has already been used with alcohol- and tobacco-related cues ^e.g.,26,30,31^, in the context of gaming and online shopping. Additionally, our study extends the sparse knowledge regarding the retest reliability of the PIT effect. Finally, we present information on the reliability and the association with symptom severity of the selectivity index, an alternative method to calculate the PIT effect by contrasting responding to two reward-related stimuli (i.e., gaming and shopping; see supplementary material). However, there are also some limitations which must be kept in mind when interpreting the results of our study. Due to recruiting mainly at two universities, our samples were highly educated, which limits the generalizability of our findings. Furthermore, our samples displayed, on average, non-problematic use of games and shopping websites. Although habit formation has been proposed as a general process in the context of reward-related behaviors, which may emerge already in early stages^60^, future studies in clinical samples are desirable. Not only must the composition of our samples but also the small sample sizes be mentioned as limitation, which could have reduced the power to detect small effects. Against this background, non-significant effects must be interpreted with caution, especially the effect of cue preference, which was based on an even smaller subsample.

There are also some limitations with regard to our PIT paradigm. Outcome insensitivity in the presence of reward-related cues might not only be explained with habitual control but might also result from attentional capture, considering that reward-related stimuli have been shown to attract attention even if irrelevant to the task at hand^61,62^. Indeed, attentional biases towards alcohol-related cues were found to mediate the impact of outcome devaluation on alcohol-related PIT effects^26^. The putative role of attentional capture does, however, not preclude the involvement of habits. During the transfer phase, reward-related cues may capture attention, thereby eliciting the sensory, albeit not motivational properties of the outcome^26^, which may then, via the S-O-R chain^9,19–22^, trigger the associated response.

Furthermore, as awareness checks were not conducted in our study, we cannot be sure if individuals successfully learned the R-O contingencies in the instrumental training and correctly understood the devaluation instruction. Given that impaired contingency knowledge has been linked to decreased devaluation effects^27^, future studies should include awareness checks to be able to control for such confounding effects. Alternatively, individuals may not lack task comprehension but display difficulties in updating instrumental beliefs and valuations. Such an effect has been observed in a study on punishment insensitivity^63^. Despite receiving explicit information about the action-outcome associations, a subgroup of participants failed to update their action-outcome beliefs, resulting in continued performance of a punished action^63^. A similar effect may underlie the post-devaluation PIT effects in our study: Despite being informed about the eliminated value of one of the outcomes, some participants might have been unable to update their action-outcome beliefs, hence continuing to choose the devalued reward. In addition to measuring such action-outcome beliefs, future studies may assess stimulus-outcome beliefs to detect whether individuals (falsely) assume that the cues presented during the transfer phase signal the availability of the associated reward.

Previous studies investigating the effect of outcome devaluation on the PIT effect have provided mixed results. One argument that has been brought forward to explain these discrepancies was the varying effectiveness of different devaluation manipulations^9,64^. A devaluation procedure that has been proven effective in reducing the PIT effect is eliminating the reward value of the outcome^65^, which made it a promising candidate to rigorously examine habit-like responding in our study. Against this background, it is even more notable that we still observed gaming- and shopping-related PIT effects after devaluation. However, it might be argued that eliminating the availability of a reward does not present a pure devaluation manipulation, which is often based on reducing the reward value, but may rather present an extinction procedure^66^. Nevertheless, as the outcome value is assumed not to be encoded in the S-O-R chain proposed by the habitual account of PIT^9,21^, habit-like responding should be insensitive not only to devaluation but also to reward extinction. Our outcome manipulation, whether it presents a devaluation or an extinction, should thus be able to differentiate between habit-like and goal-directed responding.

However, decreasing the reward value might more adequately model the phenomenon that individuals continue to engage in addictive behaviors even if the pleasure derived from it has decreased^3,67^. Possible manipulations, which reduce but not eliminate the outcome value, may include reducing the exchange value of gaming/shopping points so that they convert to a smaller voucher or confronting participants with potential negative consequences of excessive gaming/shopping, similar to the use of health warnings to devalue food^22,68^ and tobacco-related outcomes^22^.

Although we labelled the gray square used in our study as neutral stimulus, it was intended to rather reflect a no-cue condition against which to assess the influence of gaming-/shopping-related cues. Calculating the PIT effect as difference between a cue and no-cue condition is a common approach in the study of PIT effects^7^. However, as the gaming/shopping pictures differed in complexity from the gray square, they might have biased responding due to a greater salience or arousal. Although we consider this risk as small given that the gaming/shopping pictures did not increase responding in general but specifically increased responding for the congruent outcome, future studies might benefit from additionally including perceptually matched neutral pictures in the transfer phase.

Regarding the retest reliability, the time interval considered was very short, as both PIT administrations were conducted within a single laboratory session and separated by a time interval of only 30–40 minutes. Hence, more information regarding the stability of the PIT effect over days, weeks or months is needed. Furthermore, participants conducted a non-training version of an approach-avoidance task between the two measurement points of the PIT. Due to the balanced number of approach and avoidance trials, the task is unlikely to establish an approach or avoidance bias towards gaming-/shopping-related cues. Of course, we cannot rule out that the confrontation with gaming-/shopping-related stimuli enhanced craving or the desire to engage in these activities. However, when testing retest reliability over a longer time (e.g., days, weeks), participants are also likely to encounter gaming- and shopping-related stimuli between the two time points given the frequent presence of such stimuli in daily life.

### Clinical implications

Several participants in our non-clinical sample continued responding for the devalued outcome, especially when being confronted with gaming- and shopping-related cues, hence seeming to demonstrate at least partly habit-like behavior. While habits are not pathological in general^2,69^, they can become maladaptive if they concern behaviors individuals intend to change^69^. In individuals with gaming disorder or compulsive buying-shopping, habit-like responses towards addiction-related cues in combination with reduced inhibitory control^70,71^ may contribute to maintaining the problematic behavior and increase the risk of relapse.

Potential interventions to reduce the influence of addiction-related cues are approach-avoidance trainings^72,73^ or evaluative conditioning interventions^74–76^. Additionally, researchers have started to take advantage of virtual environments to change responses towards addiction-related cues^77,78^. As such computerized or virtual interventions have proved successful in reducing approach biases^72,73^ and craving^74,77^ as well as consumption^74,76,78^ and relapse^77^, they may also be able to modify addiction-related PIT effects. However, recent studies which tested the effectiveness of approach-avoidance trainings did not observe a reduction of the PIT effect after training, neither in a convenience sample^38^ nor in individuals with alcohol use disorder^37^. Hence, more research in how to change addiction-related PIT effects is needed. Regardless of the specific type of intervention, a broad range of cues should be included, as our results indicated generalization effects, with stimuli of non-favorite shopping websites or games equally stimulating responding for the respective rewards.

The finding that even individuals with mostly non-problematic use seemed to be susceptible to the influence of gaming- and shopping-related cues also stresses the relevance of early prevention. Concerning gaming disorder, preventions based on cognitive behavioral therapy^79^ or media literacy^80^ have proven to be effective in reducing symptoms of gaming disorder. Preventions for buying-shopping disorder have not been developed yet, however, may encompass psychoeducation and low-threshold advices like keeping a purchasing record or reserving products for 24 hours before completing the purchase^81^.

## Conclusion

Using a short version of the PIT paradigm with naturally rather than experimentally conditioned gaming and shopping cues, we observed PIT effects that were reduced but not abolished by devaluation. The magnitude of the PIT effects (before and after devaluation) demonstrated, on average, moderate stability across two administrations of the PIT task within a single laboratory session. Furthermore, generalization effects were found in the sense that cues of favorite and non-favorite games/shopping websites equally triggered responding for the respective reward.

## Supplementary Information

Below is the link to the electronic supplementary material.

Supplementary Material 1

## Data Availability

The dataset generated and analyzed during the current study is available from the corresponding author on reasonable request.

## References

[1] Everitt, B. J. & Robbins, T. W. Drug addiction: updating actions to habits to compulsions ten years on. *Annu. Rev. Psychol.* **67**, 23–50 (2016). 26253543 10.1146/annurev-psych-122414-033457

[2] Pool, E. R. & Sander, D. Vulnerability to relapse under stress: insights from affective neuroscience. *Swiss Med. Wkly.* **149**, 20151. 10.4414/smw.2019.20151 (2019). 10.4414/smw.2019.20151 31782510

[3] Brand, M. Can internet use become addictive? *Science* **376**, 798–799 (2022). 35587961 10.1126/science.abn4189

[4] Brand, M. et al. Which conditions should be considered as disorders in the international classification of diseases (ICD-11) designation of ‘other specified disorders due to addictive behaviors’? *J. Behav. Addict.* **11**, 150–159 (2022). 32634114 10.1556/2006.2020.00035 PMC9295220

[5] World Health Organization. International statistical classification of diseases and related health problems. Disorders due to addictive behaviours. https://icd.who.int/browse/2024-01/mms/en#499894965 (2019).

[6] World Health Organization. International statistical classification of diseases and related health problems. 6C7Y Other specified impulse control disorders. https://icd.who.int/browse/2025-01/mms/en#826065555%2Fother (2019).

[7] Cartoni, E., Balleine, B. & Baldassarre, G. Appetitive Pavlovian-instrumental Transfer: a review. *Neurosci. Biobehav Rev.* **71**, 829–848 (2016). 27693227 10.1016/j.neubiorev.2016.09.020

[8] Garbusow, M. et al. Pavlovian-to-instrumental transfer across mental disorders: a review. *Neuropsychobiology* **81**, 418–437 (2022). 35843212 10.1159/000525579

[9] Mahlberg, J. et al. Human appetitive Pavlovian-to-instrumental transfer: a goal-directed account. *Psychol. Res.* **85**, 449–463 (2021). 31720789 10.1007/s00426-019-01266-3

[10] Hogarth, L., Dickinson, A., Wright, A., Kouvaraki, M. & Duka, T. The role of drug expectancy in the control of human drug seeking. *J. Exp. Psychol. Anim. Behav. Process.* **33**, 484–496 (2007). 17924795 10.1037/0097-7403.33.4.484

[11] Hogarth, L. & Chase, H. W. Evaluating psychological markers for human nicotine dependence: tobacco choice, extinction, and Pavlovian-to-instrumental transfer. *Exp. Clin. Psychopharmacol.* **20**, 213–224 (2012). 22369668 10.1037/a0027203

[12] Manglani, H. R., Lewis, A. H., Wilson, S. J. & Delgado, M. R. Pavlovian-to-instrumental transfer of nicotine and food cues in deprived cigarette smokers. *Nicotine Tob. Res.* **19**, 670–676 (2017). 28486716 10.1093/ntr/ntx007 PMC5896487

[13] van Timmeren, T. et al. Intact corticostriatal control of goal-directed action in alcohol use disorder: A Pavlovian-to-instrumental transfer and outcome-devaluation study. *Sci. Rep.* **10**, 4949. 10.1038/s41598-020-61892-5 (2020). 32188908 10.1038/s41598-020-61892-5 PMC7087408

[14] Qin, C. et al. Enhanced Pavlovian-to-instrumental transfer in internet gaming disorder. *J. Behav. Addict.* **12**, 471–479 (2023). 37267086 10.1556/2006.2023.00023 PMC10316159

[15] Schmid, A. M. et al. Transfer from goal-directed behavior to stimulus-response habits and its modulation by acute stress in individuals with risky gaming behavior. *Sci. Rep.* **14**, 26015. 10.1038/s41598-024-73899-3 (2024). 10.1038/s41598-024-73899-3 PMC11522379 39472683

[16] Thomas, T. A. et al. Risky online buying-shopping behavior: the role of stress responsivity on the transfer from goal-directed behavior to stimulus-response habits. *J. Behav. Addict.* **14**, 1326–1342 (2025). 40900669 10.1556/2006.2025.00062 PMC12486293

[17] Vogel, V. et al. Pavlovian-to-instrumental transfer: a new paradigm to assess pathological mechanisms with regard to the use of internet applications. *Behav. Brain Res.* **347**, 8–16 (2018). 29522786 10.1016/j.bbr.2018.03.009

[18] Xu, L. et al. Pavlovian-to-instrumental transfer and outcome-devaluation effects in individuals with gaming experience. *Comput. Hum. Behav.* **155**, 108188. 10.1016/j.chb.2024.108188 (2024).

[19] Alarcón, D. E., Bonardi, C. & Delamater, A. R. Associative mechanisms involved in specific Pavlovian-to-instrumental transfer in human learning tasks. *Q. J. Exp. Psychol.* **71**, 1607–1625 (2018). 10.1080/17470218.2017.1342671 PMC6193757 28612645

[20] de Wit, S. & Dickinson, A. Associative theories of goal-directed behaviour: a case for animal–human translational models. *Psychol. Res.* **73**, 463–476 (2009). 10.1007/s00426-009-0230-6 PMC2694930 19350272

[21] Hogarth, L. Controlled and automatic learning processes in addiction. in The Routledge Handbook of Philosophy and Science of Addiction (eds Pickard, H. & Ahmed, S.) 325–338 (Routledge, 2018).

[22] Hogarth, L. & Chase, H. W. Parallel goal-directed and habitual control of human drug-seeking: implications for dependence vulnerability. *J. Exp. Psychol.* **37**, 261–276 (2011). 10.1037/a0022913 21500933

[23] Hogarth, L. et al. Extinction of cue-evoked drug-seeking relies on degrading hierarchical instrumental expectancies. *Behav. Res. Ther.* **59**, 61–70 (2014). 25011113 10.1016/j.brat.2014.06.001 PMC4119239

[24] Seabrooke, T., Hogarth, L. & Mitchell, C. J. The propositional basis of cue-controlled reward seeking. *Q. J. Exp. Psychol*. **69**, 2452–2470 (2016). 10.1080/17470218.2015.1115885 26595818

[25] Seabrooke, T., Hogarth, L., Edmunds, C. E. R. & Mitchell, C. J. Goal-directed control in Pavlovian-instrumental transfer. *J. Exp. Psychol. Anim. Learn. Cogn.* **45**, 95–101 (2019). 30604997 10.1037/xan0000191

[26] Rose, A. K., Brown, K., MacKillop, J., Field, M. & Hogarth, L. Alcohol devaluation has dissociable effects on distinct components of alcohol behaviour. *Psychopharmacol*. **235**, 1233–1244 (2018). 10.1007/s00213-018-4839-2 PMC5869941 29480437

[27] Hogarth, L. Addiction is driven by excessive goal-directed drug choice under negative affect: translational critique of habit and compulsion theory. *Neuropsychopharmacology* **45**, 720–735 (2020). 31905368 10.1038/s41386-020-0600-8 PMC7265389

[28] Steins-Loeber, S. et al. Does acute stress influence the Pavlovian-to-instrumental transfer effect? Implications for substance use disorders. *Psychopharmacol*. **237**, 2305–2316 (2020). 10.1007/s00213-020-05534-8 PMC7351872 32506233

[29] Lörsch, F. et al. The effect of individual differences on Pavlovian conditioning in specific Internet-use disorders. *Behav. Brain Res.* **476**, 115254. 10.1016/j.bbr.2024.115254 (2024). 39307287 10.1016/j.bbr.2024.115254

[30] Hogarth, L. Goal-directed and transfer-cue-elicited drug-seeking are dissociated by pharmacotherapy: evidence for independent additive controllers. *J. Exp. Psychol.* **38**, 266–278 (2012). 10.1037/a0028914 22823420

[31] Martinovic, J. et al. Electrophysiological responses to alcohol cues are not associated with Pavlovian-to-instrumental transfer in social drinkers. *PLoS ONE*. **9**, e94605. 10.1371/journal.pone.0094605 (2014). 24732090 10.1371/journal.pone.0094605 PMC3986108

[32] Staiger, P. K. & White, J. M. Cue reactivity in alcohol abusers: stimulus specificity and extinction of the responses. *Addict. Behav.* **16**, 211–221 (1991). 1776539 10.1016/0306-4603(91)90014-9

[33] Ha, J., Park, W., Park, S., Im, C. H. & Kim, L. EEG response to game-craving according to personal preference for games. *Soc. Cogn. Affect. Neurosci.* **16**, 995–1005 (2021). 33064824 10.1093/scan/nsaa131 PMC8421702

[34] Wulfert, E., Maxson, J. & Jardin, B. Cue-specific reactivity in experienced gamblers. *Psychol. Addict. Behav.* **23**, 731–735 (2009). 10.1037/a0017134 PMC2804265 20025381

[35] Garbusow, M. et al. Pavlovian-to-instrumental transfer in alcohol dependence: a pilot study. *Neuropsychobiology* **70**, 111–121 (2014). 25359491 10.1159/000363507

[36] Chen, H. et al. Susceptibility to interference between Pavlovian and instrumental control predisposes risky alcohol use developmental trajectory from ages 18 to 24. *Addict. Biol.* **28**, e13263. 10.1111/adb.13263 (2023). 36692874 10.1111/adb.13263

[37] Chen, K. et al. Automatic approach behaviors in alcohol dependence: does a cognitive bias modification training affect Pavlovian-to-Instrumental transfer effects? *Neuropsychobiology* **81**, 387–402 (2022). 36404705 10.1159/000526805

[38] Rosenthal, A., Chen, K. & Beck, A. Romanczuk-Seiferth, N. Modifying Pavlovian-to-instrumental transfer by approach avoidance training in healthy subjects: A proof of concept study. *Sci. Rep.* **13**, 10074. 10.1038/s41598-023-37083-3 (2023). 37344561 10.1038/s41598-023-37083-3 PMC10284857

[39] Hertzog, M. A. Considerations in determining sample size for pilot studies. *Res. Nurs. Health*. **31**, 180–191 (2008). 18183564 10.1002/nur.20247

[40] Janson, M. Die Top 10-Onlineshops in Deutschland [The top 10 online shops in Germany]. https://de.statista.com/infografik/642/top-10-online-shops-in-deutschland-nach-umsatz/ (2024).

[41] Statista. Beliebteste Online-Shops in Deutschland im Jahr 2024 [Most favorite online-shops in Germany in 2024]. https://de.statista.com/prognosen/999775/deutschland-beliebteste-online-shops (2024).

[42] game – Verband der deutschen Games-Branche. game Jahrescharts: Die erfolgreichsten neuen Games 2023 in Deutschland [game annual charts: The most successful new games in 2023 in Germany]. https://www.game.de/game-jahrescharts-die-erfolgreichsten-neuen-games-2023-in-deutschland/ (2024).

[43] Steam. Die Besten des Jahres 2023 - Die meistgespielten Spiele des Jahres, gemessen an der höchsten Anzahl gleichzeitiger Spieler [The best of 2023 - Most played games of the year, measured by highest number of simultaneous players]. https://store.steampowered.com/sale/BestOf2023?tab=3 (2024).

[44] Steam. Die Besten des Jahres 2023 - Die Topspiele gemessen am Bruttoumsatz [The best of 2023 - Top games measured by gross sales]. https://store.steampowered.com/sale/BestOf2023?tab=1 (2024).

[45] SullyGnome.com. Anzahl der Zuschauerstunden der meistgeschauten Video Games auf Twitch.tv im Februar 2025 (in Millionen) [Number of viewing hours of the most watched video games on Twitch.tv in February 2025 (in million)]. https://de.statista.com/statistik/daten/studie/586829/umfrage/meistgeschaute-videogames-auf-twitch/ (2024).

[46] Holmes, N. M., Marchand, A. R. & Coutureau, E. Pavlovian to instrumental transfer: a neurobehavioural perspective. *Neurosci. Biobehav Rev.* **34**, 1277–1295 (2010). 20385164 10.1016/j.neubiorev.2010.03.007

[47] Rinck, M. & Becker, E. S. Approach and avoidance in fear of spiders. *J. Behav. Ther. Exp. Psychiatry*. **38**, 105–120 (2007). 17126289 10.1016/j.jbtep.2006.10.001

[48] Müller, S. M. et al. Assessment of criteria for specific Internet-use disorders (ACSID-11): introduction of a new screening instrument capturing ICD-11 criteria for gaming disorder and other potential Internet-use disorders. *J. Behav. Addict.* **11**, 427–450 (2022). 35394924 10.1556/2006.2022.00013 PMC9295242

[49] World Health Organization. International statistical classification of diseases and related health problems. 6C51 Gaming disorder. https://icd.who.int/browse/2024-01/mms/en#1448597234 (2019).

[50] Király, O. et al. Validation of the Ten-Item Internet Gaming Disorder Test (IGDT-10) and evaluation of the nine DSM-5 Internet Gaming Disorder criteria. *Addict. Behav.* **64**, 253–260 (2017). 26632194 10.1016/j.addbeh.2015.11.005

[51] Müller, A., Trotzke, P., Mitchell, J. E., Zwaan, M. & Brand, M. The pathological buying screener: development and psychometric properties of a new screening instrument for the assessment of pathological buying symptoms. *PloS ONE*. **10**, e0141094. 10.1371/journal.pone.0141094 (2015). 26488872 10.1371/journal.pone.0141094 PMC4619303

[52] Müller, A. et al. The pathological buying screener: validation in a clinical sample. *Psychother. Psychosom. Med. Psychol.* **71**, 294–300 (2021). 33246347 10.1055/a-1303-4743

[53] Wegmann, E., Antons, S. & Brand, M. The experience of gratification and compensation in addictive behaviors: how can these experiences be measured systematically within and across disorders due to addictive behaviors? *Compr*. *Psychiatry* **117**, 152336. 10.1016/j.comppsych.2022.152336 (2022). 10.1016/j.comppsych.2022.152336 35843138

[54] R Core Team. R: A language and environment for statistical computing. https://www.R-project.org/ (2024).

[55] Bates, D., Mächler, M., Bolker, B. & Walker, S. Fitting linear mixed-effects models using lme4. *J. Stat. Softw.* **67**, 1–48 (2015).

[56] Wickham, H. *Ggplot2: Elegant Graphics for Data Analysis* (Springer, 2016).

[57] Fieller, E. C., Hartley, H. O. & Pearson, E. S. Tests for rank correlation coefficients.I.* Biometrika*. **44**, 470–481 (1957).

[58] Bishara, A. J. & Hittner, J. B. Confidence intervals for correlations when data are not normal. *Behav. Res. Methods*. **49**, 294–309 (2017). 26822671 10.3758/s13428-016-0702-8

[59] Koo, T. K. & Li, M. Y. A guideline of selecting and reporting intraclass correlation coefficients for reliability research. *J. Chiropr. Med.* **15**, 155–163 (2016). 27330520 10.1016/j.jcm.2016.02.012 PMC4913118

[60] Brand, M. et al. Current interpretations of the I-PACE model of behavioral addictions. *J. Behav. Addict.* **14**, 1–17 (2025). 40063161 10.1556/2006.2025.00020 PMC11974429

[61] Le Pelley, M. E., Pearson, D., Griffiths, O. & Beesley, T. When goals conflict with values: counterproductive attentional and oculomotor capture by reward-related stimuli. *J. Exp. Psychol. Gen.* **144**, 158–171 (2015). 25420117 10.1037/xge0000037

[62] Anderson, B. A., Laurent, P. A. & Yantis, S. Value-driven attentional capture. *Proc. Natl. Acad. Sci.* **108**, 10367–10371 (2011). 21646524 10.1073/pnas.1104047108 PMC3121816

[63] Jean-Richard-dit-Bressel, P. et al. A cognitive pathway to punishment insensitivity. *Proc. Natl. Acad. Sci.* **120**, e2221634120. 10.1073/pnas.2221634120 (2023). 37011189 10.1073/pnas.2221634120 PMC10104546

[64] Eder, A. B. & Dignath, D. Cue-elicited food seeking is eliminated with aversive outcomes following outcome devaluation. *Q. J. Exp. Psychol.* **69**, 574–588 (2016). 10.1080/17470218.2015.1062527 26089111

[65] Allman, M. J., DeLeon, I. G., Cataldo, M. F., Holland, P. C. & Johnson, A. W. Learning processes affecting human decision making: an assessment of reinforcer-selective Pavlovian-to-instrumental transfer following reinforcer devaluation. *J. Exp. Psychol. Anim. Behav. Process.* **36**, 402–408 (2010). 20658871 10.1037/a0017876

[66] Seabrooke, T., Le Pelley, M. E., Porter, A. & Mitchell, C. J. Extinguishing cue-controlled reward choice: effects of Pavlovian extinction on outcome-selective Pavlovian-instrumental transfer. *J. Exp. Psychol. Anim. Learn. Cogn.* **44**, 280–292 (2018). 29985045 10.1037/xan0000176

[67] Robinson, T. E. The neural basis of drug craving: an incentive-sensitization theory of addiction. *Brain Res. Rev.* **18**, 247–291 (1993). 8401595 10.1016/0165-0173(93)90013-p

[68] Kirsten, H., Seib-Pfeifer, L. E. & Gibbons, H. Helpless against food cues: the influence of pro- and anti-sugar videos on instrumental food-seeking behaviour in a Pavlovian-to-instrumental transfer paradigm. *Psychol. Health*. **37**, 633–657 (2021). 34101526 10.1080/08870446.2021.1907388

[69] Pierce-Messick, Z. & Corbit, L. H. Problematic eating as an issue of habitual control. *Prog Neuropsychopharmacol. Biol. Psychiatry*. **110**, 110294. 10.1016/j.pnpbp.2021.110294 (2021). 33662535 10.1016/j.pnpbp.2021.110294

[70] Müller, S. M. et al. Self-control abilities in specific types of problematic usage of the Internet: findings from clinically validated samples with neurocognitive tasks. *Am. J. Psychiatry*. **182**, 660–670 (2025). 40432342 10.1176/appi.ajp.20240486

[71] Wang, L., Tian, M., Zheng, Y., Li, Q. & Liu, X. Reduced loss aversion and inhibitory control in adolescents with internet gaming disorder. *Psychol. Addict. Behav.* **34**, 484–496 (2020). 31971427 10.1037/adb0000549

[72] Kakoschke, N., Kemps, E. & Tiggemann, M. Approach bias modification training and consumption: a review of the literature. *Addict. Behav.* **64**, 21–28 (2017). 27538198 10.1016/j.addbeh.2016.08.007

[73] Loijen, A., Vrijsen, J. N., Egger, J. I. M., Becker, E. S. & Rinck, M. Biased approach-avoidance tendencies in psychopathology: a systematic review of their assessment and modification. *Clin. Psychol. Rev.* **77**, 101825. 10.1016/j.cpr.2020.101825 (2020). 32143108 10.1016/j.cpr.2020.101825

[74] Houben, K., Schoenmakers, T. M. & Wiers, R. W. I didn’t feel like drinking but I don’t know why: the effects of evaluative conditioning on alcohol-related attitudes, craving and behavior. *Addict. Behav.* **35**, 1161–1163 (2010). 20810220 10.1016/j.addbeh.2010.08.012

[75] Măgurean, S., Constantin, T. & Sava, F. A. The indirect effect of evaluative conditioning on smoking. *J. Subst. Use*. **21**, 198–203 (2016).

[76] Tello, N., Bocage-Barthélémy, Y., Dandaba, M., Jaafari, N. & Chatard, A. Evaluative conditioning: a brief computer-delivered intervention to reduce college student drinking. *Addict. Behav.* **82**, 14–18 (2018). 29477901 10.1016/j.addbeh.2018.02.018

[77] Malbos, E. et al. Virtual reality cue exposure therapy for tobacco relapse prevention: a comparative study with standard intervention. *Psychol. Med.* **53**, 5070–5080 (2023). 35924727 10.1017/S0033291722002070 PMC10476066

[78] Metcalf, M., Rossie, K., Stokes, K., Tallman, C. & Tanner, B. Virtual reality cue refusal video game for alcohol and cigarette recovery support: summative study. *JMIR Serious Games*. **6**, e7. 10.2196/games.9231 (2018). 29661748 10.2196/games.9231 PMC5928331

[79] Lindenberg, K., Kindt, S. & Szász-Janocha, C. Effectiveness of cognitive behavioral therapy–based intervention in preventing gaming disorder and unspecified internet use disorder in adolescents: A cluster randomized clinical trial. *JAMA Netw. Open.* **5**, e2148995. 10.1001/jamanetworkopen.2021.48995 (2022). 35179587 10.1001/jamanetworkopen.2021.48995 PMC8857686

[80] Walther, B., Hanewinkel, R. & Morgenstern, M. Effects of a brief school-based media literacy intervention on digital media use in adolescents: cluster randomized controlled trial. *Cyberpsychology Behav. Soc. Netw.* **17**, 616–623 (2014). 10.1089/cyber.2014.0173 25126888

[81] Thomas, T. A. et al. Prevention approaches for compulsive buying-shopping disorder. *SUCHT* **70**, 347–356 (2024).

