# Supplementary material for: Measuring cue-elicited responding in the context of gaming and online shopping: Validity and reliability of a short Pavlovian-to-instrumental transfer paradigm
Source: Sci Rep. 2025 Dec 29;15:45657. doi: 10.1038/s41598-025-27859-0 (PMC12753825; doi:10.1038/s41598-025-27859-0)
Supplement: Supplementary file 1 — Supplementary Material 1 [file 41598_2025_27859_MOESM1_ESM.pdf]

Measuring Cue-Elicited Responding in the Context of Gaming and Online Shopping:

Validity and Reliability of a Short Pavlovian-to-Instrumental Transfer Paradigm

Supplementary Material

Results

Responding in the Presence of Gaming- and Shopping-Related Stimuli – Results of Repeated Measures ANOVAs

The effects of stimulus, devaluation, and time on response choice in the transfer phase were additionally analyzed with repeated-measures ANOVAs with stimulus, devaluation (before, after) and time (t1 = first session of the PIT task, t2 = second session of the PIT task) as within-subjects factors. Before averaging the response choice data across blocks of the transfer phase, we tested for block effects by conducting repeated-measures ANOVAs with stimulus (gaming, shopping, neutral) and block as within-subjects factors. These analyses were conducted separately for both times and the part before and after the devaluation.

For all repeated-measures ANOVAs, we report Greenhouse-Geisser (if Greenhouse-Geisser $\varepsilon < .75$) or Huynh-Feldt (if Greenhouse-Geisser $\varepsilon > .75$) adjusted degrees of freedom if the sphericity assumption was not met. Inspection of the data revealed non-normal distribution, however, the *F*-test has been shown to be relatively robust against violations of non-normality[1]. Partial eta squared (η_p^2) is reported as effect size. If significant main or interaction effects were found, post hoc analyses with Bonferroni corrected *t*-tests were conducted.

In the gaming sample, descriptive block differences were observed for the part after devaluation at the first time point. Responding for the devalued gaming reward seemed to increase after the first block (block 1: 16.9%, block 2: 20.8%, block 3: 22.4%, block 4: 21.9%), indicating a weakening of the devaluation effect over time. Similar to the gaming sample, descriptive block differences for the part after devaluation at the first time point were observed in the shopping sample, with responding for the devalued shopping reward increasing in the last block after

devaluation (block 1: 25.5%, block 2: 26.1%, block 3: 25.3%, block 4: 33.1%). However, when testing for block differences, separately for the part before and after devaluation and for both time points, a main effect of block or a block by stimulus interaction emerged neither in the gaming sample (all Fs ≤ 2.10, all ps ≥ .08) nor in the shopping sample (all Fs ≤ 2.99, all ps ≥ .08). Hence, for the subsequent analyses, we decided to average response choice across the four blocks before devaluation and across the four blocks after devaluation.

In the gaming sample, choice of the gaming-related response differed depending on which stimulus was presented ($F(1.27, 39.46) = 31.32$, $p < .001$, $\eta_p^2 = .50$). Presentation of the gaming-related stimuli significantly enhanced the choice of the gaming-related response compared to the neutral stimulus ($p < .001$) and the shopping-related stimuli ($p < .001$), indicating a gaming PIT effect (see Figure A1a). Additionally, the choice of the gaming-related response was significantly higher after the neutral stimulus was presented compared to when the shopping stimuli were presented ($p < .001$). Devaluation significantly reduced the choice of the gaming-related response ($F(1, 31) = 64.56$, $p < .001$, $\eta_p^2 = .68$) and this decrease was largest after presentation of the neutral stimulus as indicated by a significant stimulus by devaluation interaction ($F(2, 62) = 8.58$, $p < .001$, $\eta_p^2 = .22$). Even after devaluation, the choice of the gaming-related response was highest after the presentation of the gaming stimuli. Thus, a gaming PIT effect could still be observed. A closer look at the distribution of the individual data points (see Figure A1a) revealed that the devaluation effect differed considerably between participants, especially after the presentation of the gaming stimuli. While some individuals successfully refrained from choosing the gaming-related response and others, at least, reduced their choice of the gaming-related response, a third group continually chose the gaming-related response if the gaming stimuli were presented.

The pattern of response choice was comparable across the two sessions of the PIT paradigm, neither the main effect of time ($F(1, 31) = 1.32$, $p = .26$, $\eta_p^2 = .04$) nor any interaction with time was significant (all Fs ≤ 1.18, all ps ≥ .31).

The results for the shopping sample paralleled those of the gaming sample (see Figure A1b). The main effect of stimulus indicated that participants adapted their choice of the shopping-related response to the stimulus presented ($F(1.59, 47.78) = 16.58$, $p < .001$, $\eta_p^2 = .36$). The choice of the shopping-related response was highest after the presentation of the shopping-related stimuli, lower after the presentation of the neutral stimulus and lowest after the gaming-related stimuli were presented (all *p*s ≤ .007), indicating a shopping PIT effect. After devaluation, the choice of the shopping-related response was reduced ($F(1, 30) = 28.69$, $p < .001$, $\eta_p^2 = .49$). While this reduction was observed after all stimuli, it was largest in response to the neutral stimulus, as indicated by a significant stimulus by devaluation interaction ($F(2, 60) = 3.58$, $p = .03$, $\eta_p^2 = .11$). Despite the reduction in the choice of the shopping-related response, the shopping PIT effect, i.e., increased choice of the shopping-related response after presentation of the shopping stimuli, was still visible. Again, the devaluation effect differed considerably between participants (see Figure A1b), especially after the presentation of the shopping stimuli, with some individuals successfully stopping to choose the shopping-related response and others not reducing their choice of the shopping-related response.

In contrast to the results in the gaming sample, a main effect of time emerged in the shopping sample ($F(1, 30) = 4.41$, $p = .04$, $\eta_p^2 = .13$), indicating higher choice of the shopping-related response during the second PIT session.

Figure A1

Response Choice – Effect of Stimulus, Devaluation, and Time

a

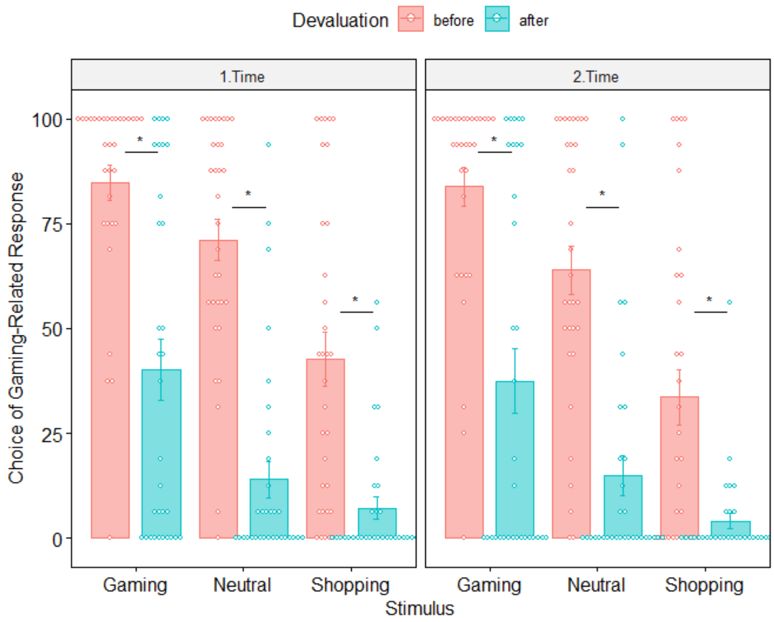

b

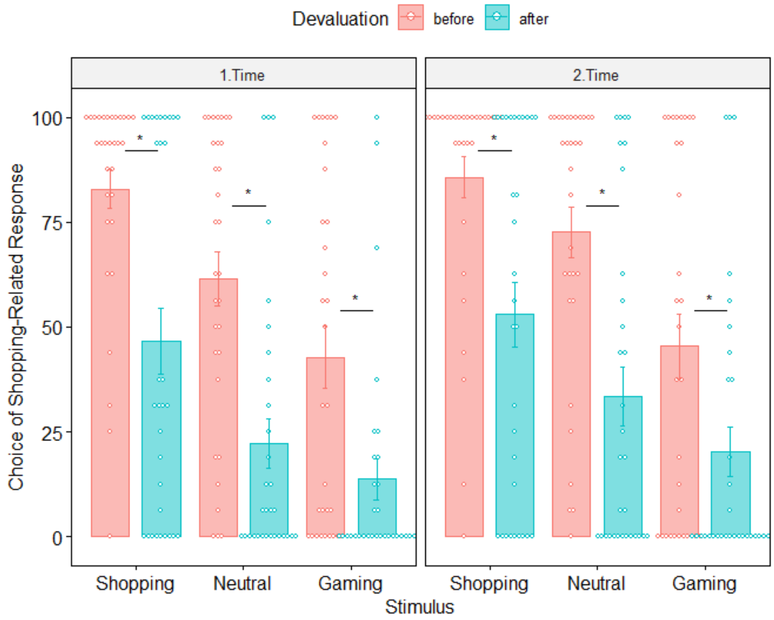

Note. Figure a: Percentage choice of the gaming-related response after presentation of the gaming-related stimuli, the neutral stimulus (gray square), and the shopping-related stimuli before and after devaluation at the first and second session of the PIT paradigm. $N = 32$. Figure b: Percentage choice of the shopping-related response after presentation of the shopping-related stimuli, the neutral

stimulus (gray square), and the gaming-related stimuli before and after devaluation at the first and second session of the PIT paradigm. $N = 31$.

Means, standard errors of the means, and individual data points are presented.

* $p < .05$

Responding in the Presence of Stimuli of Favorite Games or Shopping Websites – Results of Repeated Measures ANOVAs

We also conducted additional repeated measures ANOVAs to test whether the response choice during the transfer phase, before and after the devaluation, differed when the stimulus shown depicted a favorite game/shopping website compared to a non-favorite game/shopping website. This was tested with repeated-measures ANOVAs with preference (favorite, non-favorite) and devaluation as within-subjects factors for the first session of the PIT paradigm. Since some individuals indicated none of the games/shopping websites included in the PIT paradigm as their favorites, this analysis was conducted in reduced samples (gaming: $n = 23$; shopping: $n = 26$). As this analysis was not included in our preregistration, it presents an exploratory analysis.

Participants in the gaming sample displayed a similar preference for the gaming-related response after presentation of stimuli depicting their favorite game(s) and stimuli depicting non-favorite game(s) ($F(1, 22) = 0.01$, $p = .92$, $\eta_p^2 < .001$, see Figure A2a). Furthermore, the devaluation effect did not differ between stimuli of favorite games and stimuli of non-favorite games, as indicated by a non-significant preference by devaluation interaction ($F(1, 22) = 0.45$, $p = .51$, $\eta_p^2 = .02$). After devaluation, participants reduced their choice of the gaming-related response towards both types of stimuli, however only to some degree.

Similarly, in the shopping sample, the choice of the shopping-related response did not differ between stimuli displaying favorite shopping website(s) and stimuli depicting non-favorite shopping websites ($F(1, 25) = 1.12$, $p = .30$, $\eta_p^2 = .04$, see Figure A2b). The effect of the devaluation was comparable between both types of stimuli, as pointed out by a non-significant preference by

devaluation interaction ($F(1, 25) = 0.53$, $p = .47$, $\eta_p^2 = .02$). Hence, participants decreased their choice of the shopping-related response after devaluation regardless of whether a stimulus depicted a favorite or non-favorite shopping website.

Figure A2

Response Choice – Effect of Favorite Game/Shopping Website

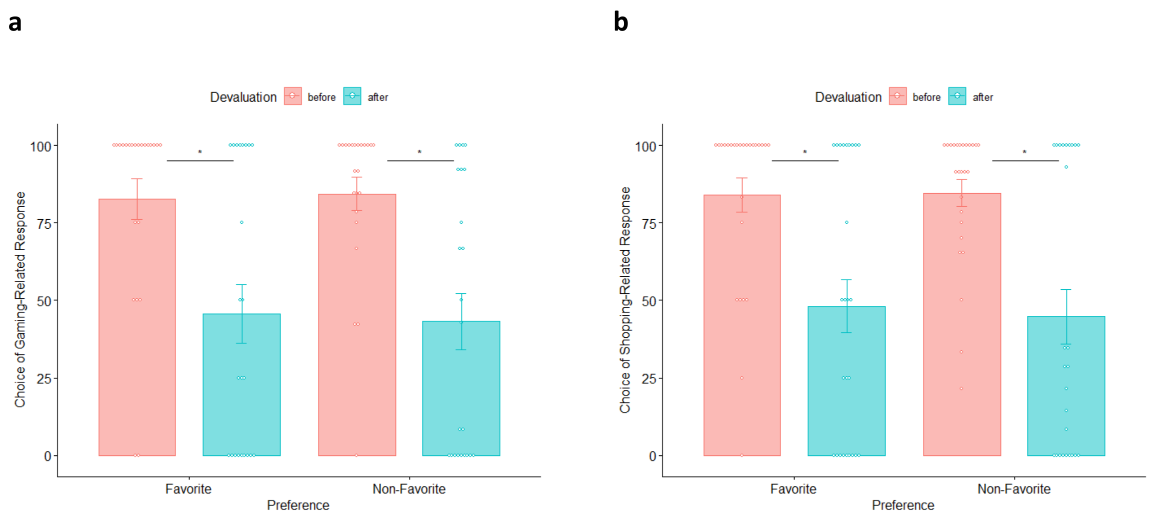

Note. Figure a: Percentage choice of the gaming-related response after presentation of stimuli depicting favorite games compared to stimuli depicting non-favorite games. $n = 23$. Figure b: Percentage choice of the shopping-related response after presentation of stimuli depicting favorite shopping websites compared to stimuli depicting non-favorite shopping websites. $n = 26$.

Means, standard errors of the means, and individual data points are presented.

* $p < .05$

Stability/Retest Reliability of the Magnitude of the Selectivity Index

In the gaming sample, the selectivity index displayed, overall, good retest reliability across two consecutive administrations of the PIT paradigm (see Table A1). Associations with symptom severity, in contrast, were non-significant and overall lower than those observed for the gaming PIT effect. Only the association between the selectivity index before devaluation and symptom severity as measured by the IGDT-10 approached significance.

Table A1

Retest Reliability and Associations with Symptom Severity for the Selectivity Index of the PIT Paradigm – Gaming Sample

	Selectivity index before devaluation	Selectivity index after devaluation
Retest reliability (relative consistency)	r_s = .88 [.77; .94], p < .001	r_s = .78 [.58; .89], p < .001
Retest reliability (absolute consistency)	ICC = .85 [.70; .92], p < .001	ICC = .79 [.61; .89], p < .001
Symptom severity gaming (IGDT-10)	r_s = .24 [-.07; 1], p = .09	r_s = .15 [-.16; 1], p = .21
Symptom severity gaming (ACSID-11 gaming)	r_s = -.08 [-.38; 1], p = .33	r_s = -.05 [-.35; 1], p = .39

Note. The selectivity index was computed as the difference between choice of the gaming-related response after the gaming-related stimuli and choice of the gaming-related response after the shopping-related stimuli. 95% confidence intervals are provided for the correlation coefficients (two-tailed for the reliability estimates and one-tailed for the associations with symptom severity). The calculation of the ICCs was based on a single measurement, absolute-agreement, 2-way mixed-effects model[2]. IGDT-10 = Ten-Item Internet Gaming Disorder Test; ACSID-11 = Assessment of Criteria for Specific Internet-Use Disorders; N = 32.

Similarly, the selectivity index in the shopping sample displayed, overall, good retest reliability (see Table A2). Associations with symptom severity, in contrast, were low and comparable to those observed for the shopping PIT effect.

Table A2

Retest Reliability and Associations with Symptom Severity for the Selectivity Index of the PIT Paradigm – Shopping Sample

	Selectivity index before devaluation	Selectivity index after devaluation
Retest reliability (relative consistency)	r_s = .66 [.39; .83], p <. 001	r_s = .77 [.56; .89], p <. 001
Retest reliability (absolute consistency)	ICC = .78 [.60; .89], p < .001	ICC = .86 [.72; .93], p < .001
Symptom severity shopping (PBS)	r_s = .01 [-.30; 1], p = .48	r_s = -.08, [-.38; 1], p = .34
Symptom severity shopping (ACSID-11 shopping)	r_s = .19 [-.13; 1], p = .16	r_s = .10 [-.21; 1], p = .29

Note. The selectivity index was computed as the difference between choice of the shopping-related response after the shopping-related stimuli and choice of the shopping-related response after the gaming-related stimuli. 95% confidence intervals are provided for the correlation coefficients (two-tailed for the reliability estimates and one-tailed for the associations with symptom severity). The calculation of the ICCs was based on a single measurement, absolute-agreement, 2-way mixed-effects model[2]. PBS = Pathological Buying Screener; ACSID-11 = Assessment of Criteria for Specific Internet-Use Disorders; *N* = 31.

References

1. Blanca, M. J., Alarcón, R., Arnau, J., Bono, R. & Bendayan, R. Non-normal data: Is ANOVA still a valid option? *Psicothema* **29**, 552–557 (2017).
2. Koo, T. K. & Li, M. Y. A guideline of selecting and reporting intraclass correlation coefficients for reliability research. *Journal of Chiropractic Medicine* **15**, 155–163 (2016).
